# Global prevalence of long COVID and its most common symptoms among healthcare workers: a systematic review and meta-analysis

**DOI:** 10.1136/bmjph-2023-000269

**Published:** 2025-04-17

**Authors:** Amani Al-Oraibi, Katherine Woolf, Jatin Naidu, Laura B Nellums, Daniel Pan, Shirley Sze, Carolyn Tarrant, Christopher A Martin, Mayuri Gogoi, Joshua Nazareth, Pip Divall, Brendan Dempsey, Danielle Lamb, Manish Pareek

**Affiliations:** 1 Respiratory Sciences, University of Leicester, Leicester, UK; 2 Lifespan and Population Health, University of Nottingham, Nottingham, UK; 3 Development Centre for Population Health, University of Leicester, Leicester, UK; 4 Research Department of Medical Education, University College London, London, UK; 5 University College London Medical School, London, UK; 6 The University of New Mexico, Albuquerque, New Mexico, USA; 7 Department of Respiratory Sciences, University of Leicester, Leicester, UK; 8 Department of Infectious Diseases and HIV Medicine, University Hospitals of Leicester NHS Trust, Leicester, UK; 9 Li Ka Shing Centre for Health Information and Discovery, University of Oxford Big Data Institute, Oxford, UK; 10 NIHR Leicester Biomedical Research Centre, Leicester, UK; 11 Cardiovascular Research Centre, Glenfield Hospital, Leicester, UK; 12 Department of Population Health Sciences, University of Leicester, Leicester, UK; 13 University Hospitals of Leicester NHS Trust, Leicester, UK; 14 Department of Applied Health Research, University College London, London, UK

**Keywords:** COVID-19, Public Health, SARS-CoV-2

## Abstract

**Objectives:**

Long COVID, a condition where symptoms persist after the acute phase of COVID-19, is a significant concern for healthcare workers (HCWs) due to their higher risk of infection. However, there is limited knowledge regarding the prevalence, symptoms and clustering of long COVID in HCWs. We aimed to estimate the pooled prevalence and identify the most common symptoms of long COVID among HCWs who were infected with SARS-CoV-2 virus globally, and investigate any differences by geographical region and other factors.

**Design:**

Systematic review and meta-analysis (PROSPERO CRD42022312781).

**Data sources:**

We searched MEDLINE, CINAHL, EMBASE, PsycINFO and the grey literature from 31 December 2019 until 18 February 2022.

**Eligibility criteria:**

We included studies reporting primary data on long COVID prevalence and symptoms in adult HCWs who had SARS-CoV-2 infection.

**Data extraction and synthesis:**

Methodological quality was assessed using the Joanna Briggs Institute checklist. Meta-analysis was performed for prevalence data of long COVID following SARS-CoV-2 infection.

**Results:**

Out of 5737 articles, 28 met the inclusion criteria, with a combined sample size of 6 481 HCWs. 15 articles scored equal to or above the median score for methodological quality. The pooled prevalence of long COVID among HCWs who had SARS-CoV-2 infection was 40% (95% CI: 29% to 51%, I^2^: 97.2%; 12 studies), with a mean follow-up period of 22 weeks. The most prevalent symptoms reported were fatigue (35%), neurologic symptoms (25%), loss/decrease of smell and/or taste (25%), myalgia (22%) and shortness of breath (19%).

**Conclusion:**

This review highlights the substantial burden of long COVID among HCWs worldwide. However, limitations in data quality and inconsistent definitions of long COVID impact the generalisability of these findings. To improve future interventions, we recommend enhanced cohort study designs for better characterisation of long COVID prevalence and symptoms in HCWs.

**WHAT IS ALREADY KNOWN ON THIS TOPIC**

- Previous evidence from studies conducted in the general population has demonstrated a high prevalence of long COVID after acute SARS-CoV-2 infection. These include a heterogeneous range of common ongoing symptoms. The global burden of long COVID in healthcare workers (HCWs) is likely to be large, when compared to the general population. However, there is limited evidence on the prevalence of long COVID in HCWs, or its symptoms and their clustering. In this systematic review and meta-analysis, we searched MEDLINE, CINAHL, EMBASE, PsycINFO and the grey literature up to February 2022 for eligible studies to synthesise evidence on the prevalence and symptoms of long COVID among HCWs infected with SARS-CoV-2 globally.

**WHAT THIS STUDY ADDS**

- This is the first systematic review and meta-analysis on the prevalence of long COVID among HCWs, to the best of our knowledge, that provides the most comprehensive review to date. No previous meta-analysis has reported symptomatology and estimated the prevalence in HCWs after SARS-CoV-2 infection. Our review shows that fatigue is the most commonly reported symptom among HCWs, with a prevalence of 35%, followed by neurologic symptoms and loss/decrease of sense of smell and/or taste (25%), myalgia (22%) and shortness of breath (19%). Additionally, we found that the pooled prevalence appears to be higher in those followed up for less than 12 months compared to those followed up for 12 months or more. Both elements combined could be fundamental in identifying the mechanistic underpinning and subsequent clinical management of long COVID among HCWs.

**HOW THIS STUDY MIGHT AFFECT RESEARCH, PRACTICE OR POLICY**

- Going forward, tools of data collection for long COVID and the definition of long COVID need harmonisation and standardisation to improve the generalisability of findings from systematic reviews of long COVID. Given the high prevalence of long COVID among HCWs, policymakers need to assess the impact of long COVID on the workforce in their areas and prioritise care for this condition. Additionally, the findings suggest the need for tailored interventions to manage workloads and ensure adequate rest for HCWs affected by long COVID. Addressing the limitations in the data, including the lack of representation from various regions, is crucial for obtaining a more accurate global perspective on the prevalence and impact of long COVID among HCWs.

## Introduction

 SARS-CoV-2 has caused significant morbidity and mortality, with over 650 million confirmed cases and more than six million deaths worldwide.^1^ Current evidence suggests that a proportion of people experience long-term symptoms that extend beyond the acute phase of infection.^2^ This is now widely known as long COVID,^3^ a debilitating phenomenon which may be unrelated to the severity of the acute infection.^4^ The National Institute for Health and Care Excellence (NICE) in England defines long COVID as ‘signs and symptoms that continue or develop after acute Coronavirus disease-2019 (COVID-19)’ that cannot be explained by another condition, and which include both ongoing symptomatic COVID-19 (from 4 to 12 weeks) and post-COVID-19 syndrome (from after 12 weeks).^5^ Typically, the symptoms of long COVID are fluctuating and most commonly involve fatigue, impaired sleep, loss of smell and taste, ‘brain fog’, shortness of breath, anxiety and depression.^6^,^8^ Nonetheless, the aetiology underlying these persistent symptoms is still unclear.^9 10^ Globally, healthcare workers (HCWs) face an elevated risk of acute SARS-CoV-2 infection compared with the general population, with prevalence of 11% confirmed by a positive SARS-CoV-2 PCR test back in 2020.^11^ HCWs, particularly those from black, Asian, Hispanic and mixed ethnicities, are at increased risk of SARS-CoV-2 infection and adverse outcomes from COVID-19.^12^ This is likely due to the numerous challenges that HCWs face while working in unprecedented circumstances, such as high work demand, a lack of personal protective equipment (PPE), long working hours and the nature of their work, given their direct and frequent contact with potentially infected patients.^13^ The increased risk of acute SARS-CoV-2 infection and long COVID for HCWs in comparison to other occupational groups is of particular concern. This is because it leads to staff shortages due to prolonged periods of sick leave, recovering and quarantining to prevent spreading of the infection.^14^ Since HCWs are likely to have greater exposure to SARS-CoV-2 infection than the general population and therefore have a higher risk of infection and subsequently a higher risk of long COVID, it is of great importance to look into this further.

HCWs’ increased exposure to the virus and the high-stress environment in which they work might contribute to a higher risk of developing long COVID. Stress and fatigue, prevalent among HCWs during the pandemic, are known to impact immune function, potentially exacerbating the risk of long-term post-viral symptoms.^15^ Additionally, the continuous exposure to COVID-19 patients and the psychological impact of the pandemic might lead to a unique trajectory of symptoms in HCWs compared with the general population.^16^

Evidence on the prevalence and risk factors of long COVID is growing and many ongoing research studies seek to understand the longterm impacts of SARS-CoV-2 infection.^17^,^19^ A recent systematic review and meta-analysis estimated the prevalence of long COVID among hospitalised and non-hospitalised COVID-19 survivors to be 45%, regardless of hospitalisation status.^20^ The estimated burden of long COVID and poor mental, physical and occupational outcomes in HCWs are likely to be large, which in turn would significantly impact the delivery of safe and highquality care. The Office for National Statistics (ONS) in the United Kingdom (UK) found that those working in health (4.47%) or social care (5.57%) had one of the highest rates of self-reported long COVID, compared with the general population (3.07%).^21^ Nonetheless, the heterogeneity of definitions used, differing follow-up time, continuous change in guidelines and lack of data regarding symptom trajectory are challenging for researchers studying long COVID.^22^ Furthermore, the prevalence of long COVID in HCWs has not been studied systematically.^23 24^ This is of urgent public health importance, since HCWs are likely to have greater exposure to COVID-19 than the general population and therefore have a higher risk of infection and subsequently a higher risk of long COVID. Moreover, there may be a higher degree of stress due to working throughout a pandemic which may exacerbate risk factors following acute infection for the development of long COVID.^25 26^

Although primary research on long COVID in general and on HCWs in particular is growing, there has been no previous comprehensive and systematic synthesis of data on the prevalence of long COVID among HCWs to inform healthcare policy, interventions, allocation of resources and future research priorities. Thus, the aim of this systematic review and meta-analysis was to estimate the pooled prevalence and identify the most common symptoms of long COVID among HCWs infected with SARS-CoV-2 virus globally, and investigate any differences by geographical region and other factors.

## Methods

This systematic review and meta-analysis followed the Preferred Reporting Items for Systematic Reviews and Meta-Analyses statement as shown in online supplemental material 1. The study protocol is published ^27^ and is registered with PROSPERO (CRD42022312781).

### Eligibility criteria

#### Condition and population

Following the NICE definition of post-COVID-19 syndrome, we defined long COVID as persistent/prolonged (constant, fluctuating or relapsing) symptoms and/or functional disability following SARS-CoV-2 infection for equal to or more than 4 weeks from onset of symptoms or from time of diagnosis, in people where SARS-CoV-2 infection was self-reported, clinically diagnosed and/or diagnosed through a laboratory test. As definitions of long COVID have varied and changed over time, we also included any study that defined/reported the condition as ‘long COVID’ or persistent symptoms following SARS-CoV-2 infection in addition to studies reporting symptoms that align with our definition (even if studies did not define it as ‘long COVID’).

HCWs are defined broadly in line with the WHO’s categorisation as all individuals engaged in actions whose primary intent is to enhance health outcomes.^28^ This includes, but is not limited to, medical, nursing and midwifery professionals; allied health professionals (such as physiotherapists, occupational therapists and pharmacists); support staff (including healthcare assistants, laundry and cleaning staff, and those involved in facility maintenance and food services); administrative and office personnel within healthcare settings; and social workers when their role directly relates to healthcare provision and patient support. This inclusive definition allows for a comprehensive analysis of global healthcare workers taking into account the variety in defining different healthcare professions worldwide, recognising the vital contributions of both patient-facing and support roles. This wide definition aligns with the definition from the International Labour Organization.^29^

#### Types of studies

Peer review supports the trustworthiness and quality of published data.^30^ Accordingly, we included all published peer-reviewed papers reporting any primary data on the prevalence of long COVID among HCWs, such as mixed-methods studies and observational study designs, including cross-sectional studies, retrospective and prospective cohort studies and case-control studies. Additionally, pre-print articles, reports and any grey literature reporting any primary quantitative data on the prevalence and/or the symptoms of long COVID following confirmed, probable or suspected SARS-CoV-2 infection were included, regardless of their peer review status as grey literature provides a more complete and comprehensive view of the available evidence.^31^

Studies such as case series or reports and qualitative study designs were excluded as these designs would not report the prevalence data required for this review.

### Search strategy

We searched the following databases from 31 December 2019 until 18 February 2022: MEDLINE (via Ovid), CINAHL (via EBSCO), EMBASE (via Ovid) and PsycINFO (via EBSCO), in addition to searching the grey literature using *MedRxiv* (pre-print server), and *OpenGrey* (grey literature database), with no language restrictions. We also manually searched websites, and reports of governmental organisations in addition to searching reference lists of eligible articles. Authors of studies were contacted to clarify missing or unclear data.

A detailed search terms and strategy table was developed for the MEDLINE database (online supplemental material 2)

### Statistical analysis

#### Meta-analysis

Eight reviewers (AA-O, JaN, DP, SS, CAM, MG, JoN) split the total number of studies equally; then, each screened titles and abstracts for eligibility using Rayyan.^32^ Then, full texts were screened by four reviewers (AA-O, JaN, DP, SS) using the same procedure. Data were extracted independently by four reviewers (AA-O, JaN, DP, SS) including citation details, study characteristics, participant characteristics relevant to the selection criteria, long COVID criteria and prevalence data. Included studies were assessed by these four reviewers for methodological quality using the Joanna Briggs Institute Critical Appraisal Checklist for Prevalence Studies.^33^ Methodological quality was assessed in relation to sampling strategy, data collection and statistical analysis. To determine the quality of individual studies, the number of ‘Yes’ responses was divided by the total number of questions to calculate the ‘% of Yes’. To examine gaps in key methodological areas across the studies, the percentage of studies that adequately met each question was calculated as ‘Total % of Yes’. This was done by dividing the number of ‘Yes’ responses for each question by the total number of studies. Studies scoring ≤33% were considered of poor quality. However, we did not exclude on the basis of quality to allow the reviewer (AA-O) to report on the extent of available evidence in this area. Disagreements were resolved by discussion between all reviewers. Each reviewer presented their perspective, and the group revisited the predefined inclusion and exclusion criteria to ensure a shared understanding.

Studies that met the inclusion criteria and reported on the prevalence of long COVID following acute COVID-19 infection among HCWs with sufficient data (ie, at least two studies) were included in the meta-analysis. A meta-analysis of the data was carried out using Stata V.16 (Stata Corp LLC, Texas, USA).^34^ Random-effects models were used to account for the variability both within and between studies.^35^ This approach was chosen because we anticipated heterogeneity due to differences in study populations, methodologies and settings. The random-effects model assumes that the true effects vary across studies, rather than being fixed, and incorporates this variation into the overall estimate.^36^ The proportion or percentage of individuals in each study sample with each long COVID symptom was pooled and 95% CIs were calculated to produce the estimated proportion across studies; a p value <0.05 was considered to be statistically significant. Where a study reported the prevalence as a percentage (% prevalence), the number of cases (n) was calculated using the following formula:


$$Number\;of\;cases\;\left(n\right)=\frac{\%\;Prevalence\times Sample\;size\;\left(N\right)}{100}$$

where (N) represents HCWs infected with COVID-19, while (n) represents HCWs with long COVID. Heterogeneity was assessed using the I^2^ statistic and explored by conducting sub-group analyses where appropriate to assess the impact of individual study characteristics on the prevalence estimates.^37^ Sensitivity analyses were carried out based on methodological quality and the most common reported symptom and by removing studies with self-reported data.^38^

Papers not reporting prevalence data or in which data could not be disaggregated for long COVID were not included in the meta-analyses. Where meta-analysis was deemed inappropriate, narrative synthesis was conducted with a descriptive summary and data tables were presented. Publication bias was assessed using Egger’s test and DOI plot for the studies included in the pooled prevalence meta-analysis.^36^ Despite the funnel plot being the most widely used plot to be used for publication bias,^39^ they have been shown to inaccurately assess publication bias in meta-analyses of proportion studies, particularly for low or high proportional outcomes.^40^ The DOI plot offers a more reliable alternative by providing a clearer visual representation of study asymmetry.^40^,^42^ Additionally, the Luis Furuya-Kanamori (LFK) index associated with the DOI plot has demonstrated superior sensitivity and diagnostic accuracy in detecting asymmetry, making it a more robust tool for bias detection in meta-analyses.^40^,^42^

We grouped symptoms according to different organ systems, based on how they were grouped in existing literature.^22 43 44^ This included the following: (1) cardiologic symptoms or diagnoses (eg, pericarditis, myocardial injury, tachycardia, syncopal attacks, chest pain), (2) fatigue, (3) shortness of breath, (4) neurologic symptoms (eg, headaches, dizziness, migraines, memory loss, insomnia or nightmares), (5) loss/decrease of sense of smell and/or taste, (6) cough, (7) alopecia, (8) myalgia (including joint pain), (9) depression, (10) anxiety, and (11) asthenia.

### Patient and public involvement

Patients and/or the public were not involved in the design, conduct, reporting or dissemination plans of this review.

### Source of funding

The funder of the study had no role in study design, data collection, data analysis, data interpretation or writing of the manuscript.

## Results

### Study selection

**Figure 1:**
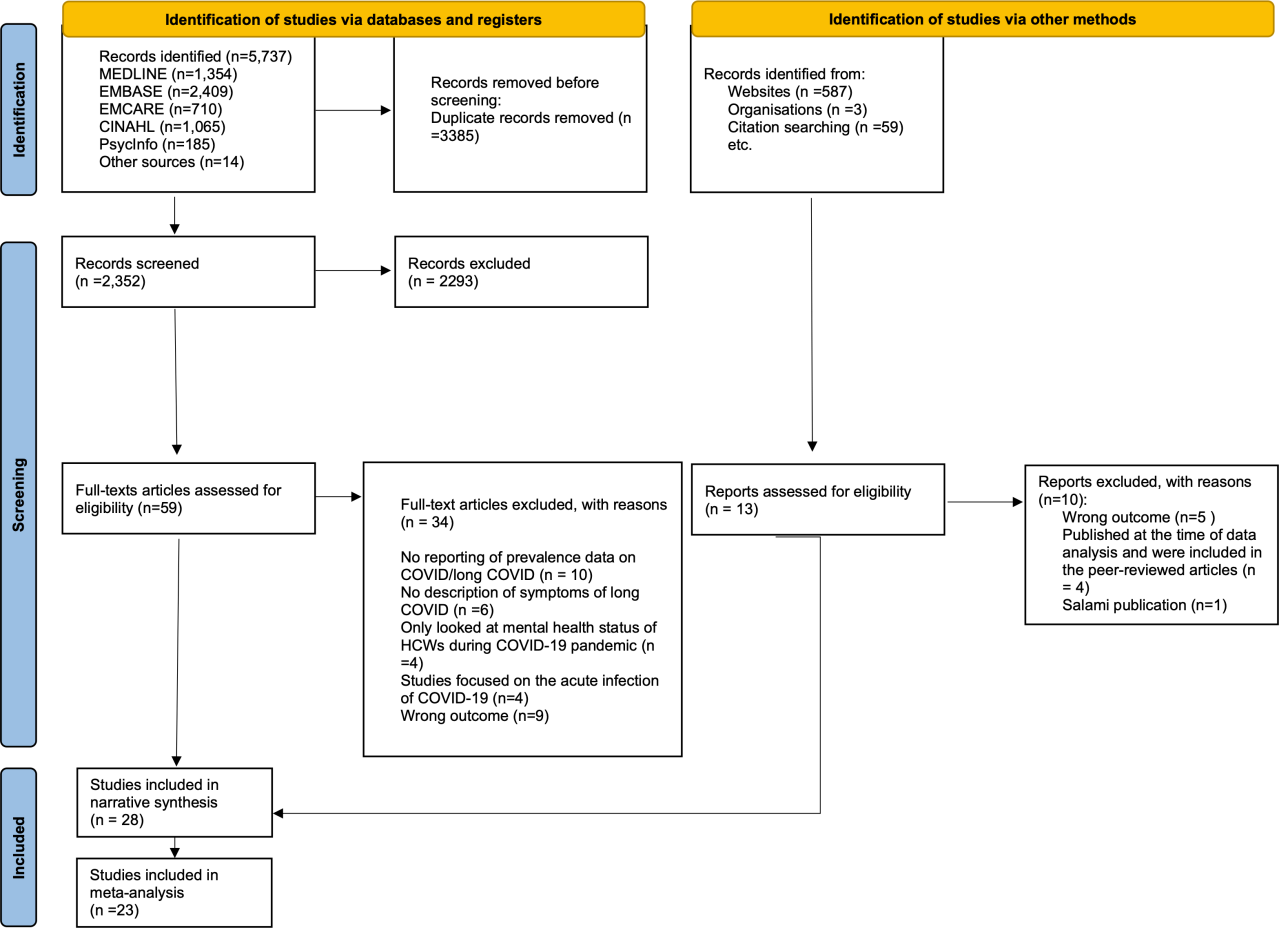
Preferred Reporting Items for Systematic Reviews and Meta-Analyses flow diagram of studies’ selection.

5737 articles were identified within the literature, between 31 December 2019 and 18 February 2022 as shown in figure 1. 3385 were duplicates and excluded. Of the 2352 remaining articles, a further 2293 were excluded after screening titles and abstracts. The remaining 59 articles were retrieved for full-text review for eligibility. A further 34 articles were excluded after fulltext review. An additional 649 records were found from other sources, including grey literature and searching citations. Of which, 13 were assessed for eligibility, where 10 were excluded and the remaining 3 articles met the inclusion criteria and were included in the review in addition to the 25 articles obtained from the database searches, giving a total of 28 articles included in the review.

Of the 28 articles included in this review, 23 articles were included in the meta-analysis.^2324 45^,^66^ The remaining five articles were included in the narrative synthesis only.^66^,^70^ The main reason for excluding articles from the meta-analysis was that some articles reported aggregated data on the prevalence of long COVID as a whole, making it difficult to identify the number of cases for each symptom of long COVID that we identified.

### Study characteristics

Online supplemental material 3 summarises the characteristics of the 28 articles that met the inclusion criteria.

A total of 6481 HCWs from 28 articles were considered in this review. Sample sizes of HCWs who have had SARS-CoV-2 infection ranged widely from 62 HCWs^58^ to 704 HCWs.^59^

The mean age of the participants was reported in 17 articles.^2446 51 53 55 56 59 60 62 63 65^,^71^ Of those articles, it ranged from 26^55^ to 45 years.^68^ Eight articles reported median age or IQR or both.^45 47 49 50 52 57 58 64^

Most of the studies included a majority of female participants, with percentages ranging from 41.8%^55^ to 92%.^54^ Only three included reported a majority of male participants with males’ prevalence ranging from 66.1%^44^ to 84.2%.^59^

13 of the 28 articles used a cross-sectional survey study design,^4851 53^,^56 58^ while 14 were cohort studies,^2445^,^47 49 50 52 57 62 63 65 68 70 71^ 8 of which were prospective,^24 45 47 49 50 57 62 70^ 5 were retrospective^46 52 65 68 71^ and one adopted a nested cohort study design.^63^ One included article was a short communication.^67^

Most studies (n=16, 57%) were from Europe; 29% (n=8) were from Asia, 7% (n=2) were from Africa and the remaining two (7%) were from North America.

22 of the 28 included articles reported on the confirmation of COVID-19 infection. 13 of these included patients who had a positive PCR test performed on either nasopharyngeal swabs or oropharyngeal swabs;^2445 46 51 57^,^59 65 67 68^ five studies included patients who had a positive SARS-CoV-2 PCR test and/or anti-nucleocapsid SARS-CoV-2 IgG antibodies test,^49 61 63 64 71^ and one study included patients who had a positive serology test (ie, antibody test).^54^ The remaining three studies had patients who were diagnosed with COVID-19 based on self-reporting.^52 60 62^

Follow-up time was reported in 24 out of 28 included studies and ranged from 42 days (6 weeks) to 491 days (~1 year and 3 months).

Only 4 of the 28 included studies reported on ethnicity.^49 58 62 63^ Two were peer-reviewed;^49 58^ one reported three categories (African, Caucasian and coloured)^58^ and the other classified ethnicity data into two categories (Caucasian and other).^49^ Two articles were pre-prints that reported more specific categories of ethnicity^62 63^ (table 1).

**Table 1:** A Summary of the prevalence for the most common symptoms of long COVID

Type of long COVID symptom	Number of included studies	Pooled prevalence estimate (95% CIs)	I^2^ statistic
Fatigue	14	35% (15 to 56)	99.6%
Neurologic	16	25% (15 to 34)	98.6%
Loss/decrease of sense of smell and/or taste	17	25% (15 to 35)	99.1%
Myalgia	12	22% (12 to 32)	99.4%
Shortness of breath	13	19% (3 to 36)	99.8%
Depression	5	18% (7 to 29)	94.6%
Cardiologic	8	17% (10 to 24)	98.1%
Asthenia	3	15% (3 to 27)	91.8%
Anxiety	6	9% (4 to 15)	91.9%
Alopecia	8	7% (3 to 11)	93.7%
Cough	6	5% (3 to 8)	82.1%

Following quality assessment, the median score for the number of ‘Yes’ in each included study is 78% (online supplemental material 4). 15 articles scored equal to or above the median score with scores ranging from 78 to 89% (online supplemental material 4). Two studies were of poor quality.^51 68^

### Prevalence

**Figure 2:**
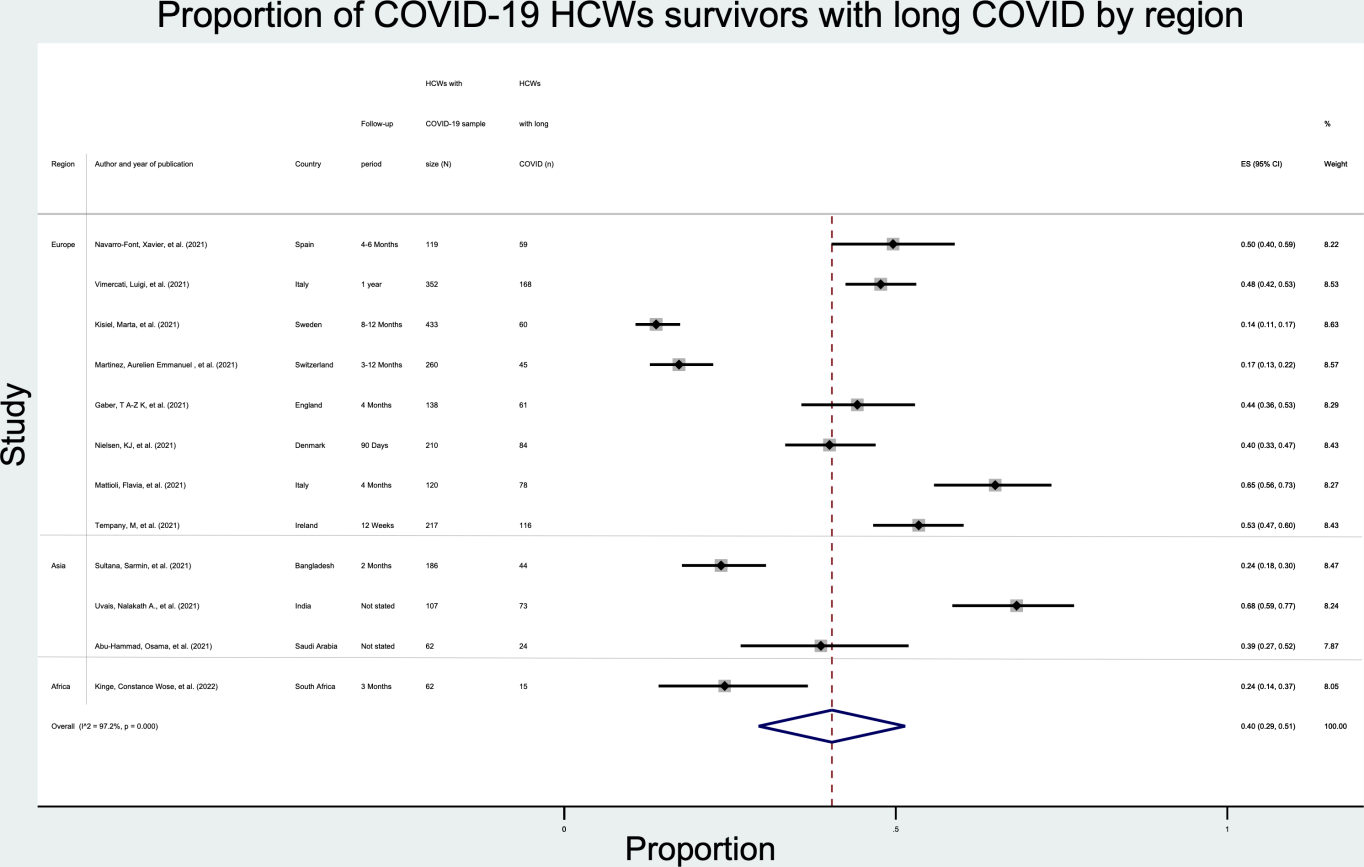
Forest plot showing point estimates with 95% CIs for the pooled prevalence of long COVID symptoms among COVID-19 HCWs survivors. HCWS, healthcare workers.

The pooled prevalence of long COVID among HCWs who had SARS-CoV-2 infection was 40% (95 CIs 29%–51%, I^2^: 97.2%; 12 studies) with a mean follow-up period of 22.2 weeks for studies that stated the follow-up time as shown in figure 2.

There were no differences in the prevalence by region as shown in online supplemental material 5.

**Figure 3:**
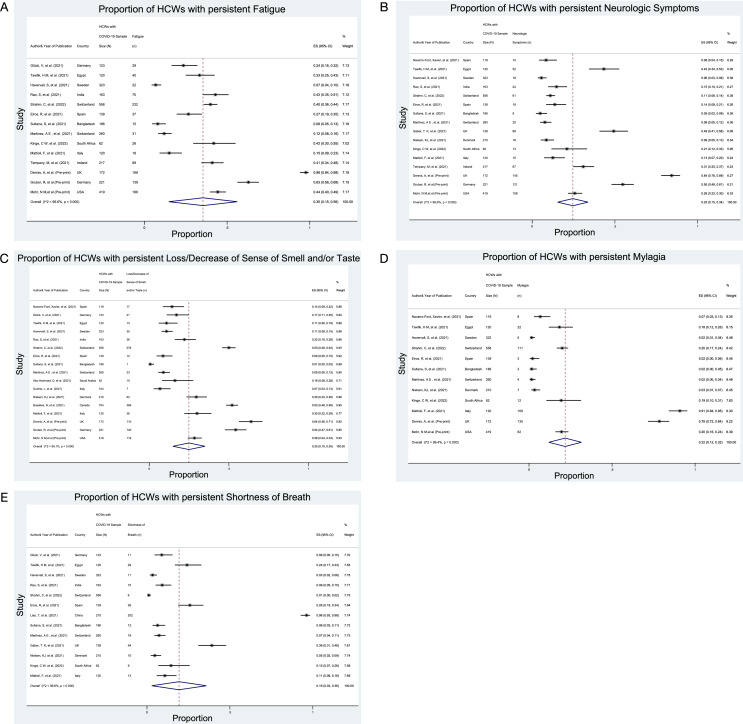
Forest plot showing point estimates with 95% CIs for prevalence of the five most common long COVID symptoms among COVID-19 HCWs survivors. HCWs, healthcare workers.

The five most prevalent symptoms reported were fatigue (35%; 95% Cl 15%–56%; 14 studies), neurologic symptoms (25%; 95% CI 15% to 34%; 16 studies), loss/decrease of sense of smell and/or taste (25%; 95% CI 15% to 35%; 17 studies), myalgia (22%; 95% CI 12%–32%; 12 studies) and shortness of breath (19%; 95% CI 3% to 36%; 13 studies) as shown in figure 3. A summary of the prevalence estimates of the meta-analyses for the 11 most common symptoms of long COVID among HCWs infected with SARS-CoV-2 virus is presented in table 1. Forest plots of the remaining six symptoms are presented in supplementary materials (online supplemental material 6).

The estimated pooled prevalence appeared lower in studies with follow-up period ≥12 months (estimated pooled prevalence: 26%; 95% Cl: 7%–46%: three studies) compared with those with <12 months of follow-up (estimated pooled prevalence: 43%; 95% Cl: 32%–54%: seven studies); see online supplemental material 7.

Sensitivity analyses of studies that were rated as acceptable quality and peer reviewed showed no changes in the estimated prevalence (online supplemental material 8). Furthermore, sensitivity analyses of the pooled prevalence of the most common symptoms, as well as only studies where data were not self-reported, showed no differences compared with overall pooled prevalence of long COVID (online supplemental materials 8 and 9).

The DOI plot indicates a significant asymmetry in the data (number of included studies=12), suggesting the presence of publication bias (Egger’s test, p=0.011, LFK index=3.63; online supplemental material 10). These 12 studies were included in the DOI plot as they formed the basis of our pooled prevalence meta-analysis.

## Discussion

This paper reports the first meta-analysis of the global prevalence of long COVID among HCWs who had SARS-CoV-2 infection. Overall, the meta-analyses showed a high prevalence of long COVID among HCWs who had COVID-19 (40%) within a follow-up range from 42 to 491 days. Compared with the general population, existing systematic reviews and meta-analyses estimated a prevalence of 43%^72^ and 45%,^20^ which appears similar or slightly higher than among HCWs. However, these comparisons must consider significant methodological differences between the studies. First of all, both reviews on the general population used broader inclusion criteria and earlier follow-up definitions,^20 72^ which may partially explain differences in prevalence estimates. For example, O’Mahoney *et al* included individuals with ongoing symptoms for a minimum of 28 days, aligning with ONS data and focusing on COVID-19 survivors with at least 100 participants per study.^20^ Their mean follow-up period was approximately 126 days.^20^ Chen *et al* defined post-COVID-19 condition as symptoms at least 28 days after infection and analysed outcomes at various time points (30, 60, 90 and 120 days).^72^ While this approach aligns with WHO definitions at later stages, its broader inclusion criteria likely skewed prevalence estimates by including transient or short-term symptoms. In contrast, our review aligned with the NICE definition (symptoms persisting 4+ weeks) but focused on HCWs and followed participants for a mean of 22 weeks (42–491 days). Furthermore, subgroup analyses indicated that follow-up duration affects prevalence estimates: studies with follow-up periods <12 months reported a pooled prevalence of 43%, compared with 26% in those with follow-up ≥12 months. This variability in prevalence estimates may also stem from differences in definitions and timepoints of assessment, as noted by Chen *et al*.^73^ The lack of standardisation in health outcome measures and long COVID diagnostic criteria across studies further complicates comparisons.^74 75^ Second, the general population reviews combined hospitalised and non-hospitalised individuals, which can skew prevalence estimates due to the higher symptom burden often associated with hospitalisation. Notably, Chen *et al*’s review reported a prevalence of 33% among non-hospitalised individuals.^72^ This suggests that hospitalisation status influences prevalence estimates. Finally, the differences in long COVID prevalence between HCWs in my findings and the general population in existing literature could be explained by the health-seeking behaviours of HCWs as they might neglect their long COVID symptoms as they might consider them ‘non-specific’, thereby affecting the accurate reporting of long COVID.^76^

We also found that the most commonly reported symptom was fatigue (35%), followed by neurologic symptoms and loss/decrease of sense of smell and/or taste (25%), myalgia (22%) and shortness of breath (19%). These findings align with recent evidence showing fatigue as the most prevalent long COVID symptom.^20 77 78^ However, differences in symptom prevalence, such as the higher reporting of pain/discomfort (27.9%) in hospitalised patients,^20^ reflect variations in initial illness severity, as hospitalised patients often report higher levels of pain due to more severe acute infections.^20 79 80^ HCWs face unique stressors, such as long and irregular working hours, high job demands, and emotional stress, which may exacerbate symptoms like myalgia and sleep disturbances.^81 82^ Evidence suggests that extended work shifts and lack of sleep increase the risk of errors,^83^ posing a significant threat to patient safety. Healthcare institutions should consider limiting shift lengths, managing workloads and providing adequate rest periods to mitigate these effects.^84^

This is the first systematic review and meta-analysis to estimate the prevalence of long COVID among HCWs globally. A key strength is that this systematic review and meta-analysis identified the most common symptoms of long COVID in this population using robust methods. We also registered the review’s protocol with PROSPERO and published it for enhancing transparency of the review. The high levels of heterogeneity were expected as with similar systematic reviews and meta-analyses^20 22 85^ owing to varied sample sizes, settings/contexts, study country and different follow-up periods. In addition, the evolving nature of long COVID, including its definition and diagnostic criteria, presents a significant limitation. At the time of our literature search (December 2019 to February 2022), long COVID was a relatively new and evolving concept, with no universally accepted definition or diagnostic criteria. This variability could affect the comparability and generalisability of the findings across studies. As such, the pooled prevalence estimates should be interpreted with caution. Nonetheless, we explored the heterogeneity and attempted to minimise it by conducting subgroup analysis by follow-up period, and region. The subgroup analysis by follow-up period provides insight into the difference in prevalence estimates. Studies with follow-up periods of <12 months reported a higher pooled prevalence (43%) compared with those with follow-up periods of ≥12 months (26%). This aligns with existing literature as a longitudinal study showed that a significant proportion of patients reported persistent symptoms at early follow-up stages, which reduced substantially by 15 months.^86^ Another study by the Centers for Disease Control and Prevention(CDC) reported that the prevalence of symptoms declined substantially from baseline to 3-month follow-up and continued to decrease at 6-month, 9-month and 12-month follow-ups.^87^ One potential reason for this is that early follow-up may capture more acute symptoms or immediate effects of an exposure, which might decrease or resolve over time.^88^ Another reason could be that longer follow-up periods may allow for a greater degree of recovery or adaptation, leading to a lower observed prevalence over time.^86^ This subgroup analysis suggests that the duration of follow-up is influencing the reported prevalence of long COVID symptoms. Longer follow-up periods might capture recovery or improvement in symptoms, thereby resulting in lower prevalence estimates. However, our results should be interpreted with caution due to the small number of studies (n = 3) in the ≥12-month follow-up group, which might limit the credibility of the observed significant difference.

We also conducted different sensitivity analyses to assess the impact of the quality of the included studies. The sensitivity analyses indicated no significant change in the pooled prevalence estimates. This suggests that the estimated prevalence at 40% is likely to be an accurate estimate and that the inclusion of lower-quality studies did not substantially affect the overall prevalence estimates. These findings underscore the consistency of our prevalence estimates despite the high levels of heterogeneity, indicating that the observed variability is not solely attributable to the quality of the studies included.

While sensitivity analyses confirmed consistency in our prevalence estimates, the lack of detailed ethnicity-disaggregated data limited further subgroup exploration. We performed a thorough search of the literature using a range of electronic databases, including grey literature search and screened reference lists of full texts and previous reviews without restriction on the language, but the possibility remains that we may have missed recently published eligible studies. Nonetheless, publication bias was assessed using a DOI plot which suggested the presence of publication bias. This bias may have resulted from smaller studies with non-significant or negative results being less likely to be published or indexed in searchable databases. Consequently, our results should be interpreted with caution, considering the potential influence of publication bias. Our findings provide critical insights into the prevalence and symptoms of long COVID among HCWs, a group at high risk due to their occupational exposure and stress. The high prevalence (40%) raises concerns about the impact on healthcare services, as prolonged symptoms among HCWs may exacerbate workforce shortages and compromise patient care. Addressing these challenges requires exploring disparities in symptom profiles across ethnic groups, given known inequities in COVID-19 outcomes influenced by factors such as socioeconomic status and pre-existing conditions. Understanding these variations is essential to reducing health inequities and informing tailored interventions. Furthermore, identifying the mechanisms underlying long COVID is imperative to enable early management, prevent long-term complications, and support HCWs in returning to their professional and personal lives. These findings underscore the importance for policymakers and healthcare systems to prioritise support and resources for HCWs affected by long COVID to safeguard the stability of healthcare services and patients’ safety.

## Conclusion

Our findings provide an insight into the burden of long COVID among HCWs who had SARS-CoV-2 infection. Given the relatively high prevalence (40%), healthcare services and policy need to prioritise long COVID care among HCWs. To ensure consistent prevalence estimates of long COVID, it is crucial to develop standardised definitions and diagnostic criteria that can be uniformly applied across studies. Additionally, encouraging the publication of all research findings, including those with non-significant results, is essential to mitigate the impact of publication bias, which can skew the overall understanding of long COVID prevalence. Future studies should be designed with longer follow-up periods to gain deeper insights into the trajectory of long COVID symptoms over time. Given that fatigue, neurologic symptoms and myalgia were identified as the most prevalent symptoms among HCWs in our review, prioritising interventions that target these symptoms is vital for improving their quality of life. Furthermore, regional factors contributing to variations in prevalence should be investigated to inform region-specific healthcare policies and support systems, ensuring that the needs of HCWs in different contexts are adequately addressed.

## Supplementary material

10.1136/bmjph-2023-000269 online supplemental material 1

## Data Availability

Data sharing not applicable as no datasets generated and/or analysed for this study.
