# Supplementary material for: Global prevalence of long COVID and its most common symptoms among healthcare workers: a systematic review and meta-analysis
Source: BMJ Public Health. 2025 Apr 17;3(1):e000269. doi: 10.1136/bmjph-2023-000269 (PMC12010341; doi:10.1136/bmjph-2023-000269)

Supplementary Materials | Global prevalence of long COVID and its most common symptoms among healthcare workers: a systematic review and meta-analysis (SUPPLEMENTARY MATERIALS)

Supplemental Material 1. PRISMA 2020 Checklist

Section and Topic	Item #	Checklist item	Location where item is reported
**TITLE**			
Title	1	Identify the report as a systematic review.	1
**ABSTRACT**			
Abstract	2	See the PRISMA 2020 for Abstracts checklist.	1&2
**INTRODUCTION**			
Rationale	3	Describe the rationale for the review in the context of existing knowledge.	4
Objectives	4	Provide an explicit statement of the objective(s) or question(s) the review addresses.	5
**METHODS**			
Eligibility criteria	5	Specify the inclusion and exclusion criteria for the review and how studies were grouped for the syntheses.	5
Information sources	6	Specify all databases, registers, websites, organisations, reference lists and other sources searched or consulted to identify studies. Specify the date when each source was last searched or consulted.	5
Search strategy	7	Present the full search strategies for all databases, registers and websites, including any filters and limits used.	Supplementary materials
Selection process	8	Specify the methods used to decide whether a study met the inclusion criteria of the review, including how many reviewers screened each record and each report retrieved, whether they worked independently, and if applicable, details of automation tools used in the process.	6
Data collection process	9	Specify the methods used to collect data from reports, including how many reviewers collected data from each report, whether they worked independently, any processes for obtaining or confirming data from study investigators, and if applicable, details of automation tools used in the process.	6
Data items	10a	List and define all outcomes for which data were sought. Specify whether all results that were compatible with each outcome domain in each study were sought (e.g. for all measures, time points, analyses), and if not, the methods used to decide which results to collect.	6
	10b	List and define all other variables for which data were sought (e.g. participant and intervention characteristics, funding sources). Describe any assumptions made about any missing or unclear information.	6
Study risk of bias assessment	11	Specify the methods used to assess risk of bias in the included studies, including details of the tool(s) used, how many reviewers assessed each study and whether they worked independently, and if applicable, details of automation tools used in the process.	6
Effect measures	12	Specify for each outcome the effect measure(s) (e.g. risk ratio, mean difference) used in the synthesis or presentation of results.	6
Synthesis methods	13a	Describe the processes used to decide which studies were eligible for each synthesis (e.g. tabulating the study intervention characteristics and comparing against the planned groups for each synthesis (item #5)).	6
	13b	Describe any methods required to prepare the data for presentation or synthesis, such as handling of missing summary statistics, or data	Not applicable

Section and Topic	Item #	Checklist item	Location where item is reported
		conversions.	
	13c	Describe any methods used to tabulate or visually display results of individual studies and syntheses.	6
	13d	Describe any methods used to synthesize results and provide a rationale for the choice(s). If meta-analysis was performed, describe the model(s), method(s) to identify the presence and extent of statistical heterogeneity, and software package(s) used.	6
	13e	Describe any methods used to explore possible causes of heterogeneity among study results (e.g. subgroup analysis, meta-regression).	6
	13f	Describe any sensitivity analyses conducted to assess robustness of the synthesized results.	6
Reporting bias assessment	14	Describe any methods used to assess risk of bias due to missing results in a synthesis (arising from reporting biases).	6
Certainty assessment	15	Describe any methods used to assess certainty (or confidence) in the body of evidence for an outcome.	Not applicable
**RESULTS**			
Study selection	16a	Describe the results of the search and selection process, from the number of records identified in the search to the number of studies included in the review, ideally using a flow diagram.	7
	16b	Cite studies that might appear to meet the inclusion criteria, but which were excluded, and explain why they were excluded.	7 and supplementary materials
Study characteristics	17	Cite each included study and present its characteristics.	12 – 23
Risk of bias in studies	18	Present assessments of risk of bias for each included study.	12 – 23 & supplementary materials
Results of individual studies	19	For all outcomes, present, for each study: (a) summary statistics for each group (where appropriate) and (b) an effect estimate and its precision (e.g. confidence/credible interval), ideally using structured tables or plots.	24 & supplementary materials
Results of syntheses	20a	For each synthesis, briefly summarise the characteristics and risk of bias among contributing studies.	8&9
	20b	Present results of all statistical syntheses conducted. If meta-analysis was done, present for each the summary estimate and its precision (e.g. confidence/credible interval) and measures of statistical heterogeneity. If comparing groups, describe the direction of the effect.	25 & supplementary materials
	20c	Present results of all investigations of possible causes of heterogeneity among study results.	
	20d	Present results of all sensitivity analyses conducted to assess the robustness of the synthesized results.	supplementary materials
Reporting biases	21	Present assessments of risk of bias due to missing results (arising from reporting biases) for each synthesis assessed.	NA
Certainty of evidence	22	Present assessments of certainty (or confidence) in the body of evidence for each outcome assessed.	NA

Section and Topic	Item #	Checklist item	Location where item is reported
**DISCUSSION**			
Discussion	23a	Provide a general interpretation of the results in the context of other evidence.	9&10
	23b	Discuss any limitations of the evidence included in the review.	10
	23c	Discuss any limitations of the review processes used.	10
	23d	Discuss implications of the results for practice, policy, and future research.	10
**OTHER INFORMATION**			
Registration and protocol	24a	Provide registration information for the review, including register name and registration number, or state that the review was not registered.	1
	24b	Indicate where the review protocol can be accessed, or state that a protocol was not prepared.	1
	24c	Describe and explain any amendments to information provided at registration or in the protocol.	NA
Support	25	Describe sources of financial or non-financial support for the review, and the role of the funders or sponsors in the review.	2
Competing interests	26	Declare any competing interests of review authors.	24
Availability of data, code and other materials	27	Report which of the following are publicly available and where they can be found: template data collection forms; data extracted from included studies; data used for all analyses; analytic code; any other materials used in the review.	NA

Supplemental Material 2.. Search Terms and Strategy for MEDLINE (via Ovid)

MEDLINE (via Ovid) *from 2019 to 18 Feb 2022*

Search ID #	Search terms	Results
**1**	(longcovid* or long covid* or longcoronavirus* or longcorona* virus* or long coronavirus* or long corona* virus* or longCov or long Cov or longsars* or long sars* or "long severe acute respiratory syndrome*" or longncov* or long ncov*).ti,ab,kw,kf.	929
**2**	((long* or endur* or legacy* or slow* or gradual* or protract* or lengthy* or chronic* or persist* or relaps* or remit* or remission* or residual* or delay* or prolong* or extend* or linger* or permanent* or fluctuat* or sequela* or multisystem* or "multi system*" or nonrecover* or "non recover*" or subacute* or "sub acute*" or lasting* or continuous* or continual* or continuing* or postacute* or "post acute*" or postdischarg* or "post discharg*" or postinfect* or "post infect*" or postviral* or "post viral*" or postvirus* or "post	1,331

	virus*") adj1 (covid* or coronavirus* or corona* virus* or Cov or "SARS-CoV-2*" or "SARSCoV-2*" or "SARSCoV2*" or "SARS-CoV2*" or "severe acute respiratory syndrome*" or Ncov* or "n-cov")).ti,ab.	
**3**	(("long* term*" or longterm* or "long* haul*" or longhaul* or "long* tail*" or longtail* or longduration* or "long duration*" or longlast* or "long last*" or longstanding* or "long standing*" or "medium* term*" or mediumterm*) adj3 (covid* or coronavirus* or corona* virus* or Cov or "SARS-CoV-2*" or "SARSCoV-2*" or "SARSCoV2*" or "SARS-CoV2*" or "severe acute respiratory syndrome*" or Ncov* or "n-cov")).ti,ab.	980
**4**	((postcovid* or post covid* or postcoronavirus* or postcorona* virus* or post coronavirus* or post corona* virus* or postcoronavirinae* or postcorona* virinae* or post coronavirinae* or post corona* virinae* or postCov or post Cov or postsars* or post sars* or "post severe acute respiratory syndrome*" or postncov* or post ncov*) adj3 (syndrome* or disorder* or illness* or sickness* or disease* or condition* or symptom* or sign* or prognos* or followup* or "follow up*" or feature* or comorbid* or "co morbid*" or multimorbid* or "multi morbid*" or survivor* or survival* or risk* or care* or convalescen* or recuperat* or aftercare* or ambulatory* or outpatient* or "out patient*")).ti,ab.	765
**5**	((ongoing* or long* or endur* or legacy* or slow* or gradual* or protract* or lengthy* or chronic* or persist* or relaps* or remit* or remission* or residual* or delay* or prolong* or extend* or linger* or permanent* or fluctuat* or multisystem* or "multi system*" or nonrecover* or "non recover*" or subacute* or "sub acute*" or lasting* or continuous* or continual* or continuing* or postacute* or "post acute*" or postdischarg* or "post discharg*" or postinfect* or "post infect*" or postviral* or "post viral*" or postvirus* or "post virus*" or "medium* term*" or mediumterm*) adj4 (sequela* or illness* or symptom* or sign* or prognos* or rehab* or convalescen* or recuperat* or followup* or "follow up*" or feature*) adj10 (covid* or coronavirus* or corona* virus* or Cov or "SARS-CoV-2*" or "SARSCoV-2*" or "SARSCoV2*" or "SARS-CoV2*" or "severe acute respiratory syndrome*" or Ncov* or "n-cov")).ti,ab.	2,369
**6**	((ongoing* or long* or endur* or legacy* or slow* or gradual* or protract* or lengthy* or chronic* or persist* or relaps* or remit* or remission* or residual* or delay* or prolong* or extend* or linger* or permanent* or fluctuat* or multisystem* or "multi system*" or subacute* or "sub acute*" or lasting* or continuous* or continual* or continuing* or post* or after* or follow* or "medium* term*" or mediumterm*) adj1 recover* adj10 (covid* or coronavirus* or corona* virus* or Cov or "SARS-CoV-2*" or "SARSCoV-2*" or "SARSCoV2*" or "SARS-CoV2*" or "severe acute respiratory syndrome*" or Ncov* or "n-cov")).ti,ab.	687
**7**	or/1-6	4,958
**8**	exp coronavirus/	126,181

**9**	exp Coronavirus Infections/	152,493
**10**	(covid* or coronavirus* or corona* virus* or Cov or "SARS-CoV-2*" or "SARSCoV-2*" or "SARSCoV2*" or "SARS-CoV2*" or "severe acute respiratory syndrome*" or Ncov* or "n-cov").ti.	208,852
**11**	or/8-10	234,542
**12**	"Recovery of Function"/	57,919
**13**	Aftercare/	11,282
**14**	rehabilitation/	18,638
**15**	Activities of Daily Living/	69,497
**16**	convalescence/	3,879
**17**	convalescence/	789

**18**	Physical Functional Performance/	2,220
**19**	Physical Functional Performance/	159,088
**20**	11 and 19	1,037
**21**	("long* haul*" or longhaul* or "long* tail*" or longtail* or longduration* or "long duration*" or longlast* or "long last*" or longstanding* or "long standing*").ti,ab. and (8 or 9)	718
**22**	((recover* or nonrecover*) adj3 function* adj10 (covid* or coronavirus* or corona* virus* or Cov or "SARS-CoV-2*" or "SARSCoV-2*" or "SARSCoV2*" or "SARS-CoV2*" or "severe acute respiratory syndrome*" or Ncov* or "n-cov")).ti,ab.	80
**23**	((postacute* or "post acute*" or postdischarg* or "post discharg*" or postinfect* or "post infect*" or postviral* or "post viral*" or postvirus* or "post virus*" or subacute* or "sub acute*") adj3 (care* or convalescen* or recuperat* or aftercare* or ambulatory* or outpatient* or "out patient*" or survivor* or survival*) adj10 (covid* or coronavirus* or corona* virus* or Cov or "SARS-CoV-2*" or "SARSCoV-2*" or "SARSCoV2*" or "SARS-CoV2*" or "severe acute respiratory syndrome*" or Ncov* or "n-cov")).ti,ab.	68
**24**	((convalescen* or recuperat* or after* or followup* or "follow up*" or rehab*) adj1 (therap* or care*) adj10 (covid* or coronavirus* or corona* virus* or Cov or "SARS-CoV-2*" or "SARSCoV-2*" or "SARSCoV2*" or "SARS-CoV2*" or "severe acute respiratory syndrome*" or Ncov* or "n-cov")).ti,ab.	220
**25**	((ongoing* or endur* or long* or legacy* or slow* or gradual* or protract* or lengthy* or chronic* or persist* or relaps* or remit* or remission* or residual* or delay* or prolong* or extend* or linger* or permanent* or fluctuat* or multisystem* or "multi system*" or nonrecover* or "non recover*" or subacute* or "sub acute*" or lasting* or continuous* or continual* or continuing* or postacute* or "post acute*" or postdischarg* or "post discharg*" or postinfect* or "post infect*" or postviral* or "post viral*" or postvirus* or "post virus*" or "medium* term*" or mediumterm* or adverse* or dangerous* or harmful* or indirect* or injurious* or secondary* or side effect* or undesirable* or sequela* or complication* or consequence* or effect* or event* or impact* or outcome* or reaction* or complexit* or aftercare* or impair* or problem* or issue* or rehab* or function* or perform*) adj10 ((daily* or everyday* or day* or	422

	normal* or usual*) adj1 (activit* or living* or life* or lives* or job* or work* or employ* or occupation* or hobby* or hobbies* or leisure*)) adj10 (covid* or coronavirus* or corona* virus* or Cov or "SARS-CoV-2*" or "SARSCoV-2*" or "SARSCoV2*" or "SARS-CoV2*" or "severe acute respiratory syndrome*" or Ncov* or "n-cov")).ti,ab.	
**26**	((ongoing* or endur* or long* or legacy* or slow* or gradual* or protract* or lengthy* or chronic* or persist* or relaps* or remit* or remission* or residual* or delay* or prolong* or extend* or linger* or permanent* or fluctuat* or multisystem* or "multi system*" or nonrecover* or "non recover*" or subacute* or "sub acute*" or lasting* or continuous* or continual* or continuing* or postacute* or "post acute*" or postdischarg* or "post discharg*" or postinfect* or "post infect*" or postviral* or "post viral*" or postvirus* or "post virus*" or "medium* term*" or mediumterm*) adj3 ((health* or adverse* or dangerous* or harmful* or indirect* or injurious* or secondary* or side* or undesirable* or negative* or damaging* or detriment* or abnormal*) adj3 (sequela* or complication* or consequence* or effect* or event* or impact* or outcome* or reaction* or complexit* or aftercare* or impair* or problem* or issue* or symptom* or disorder*)) adj10 (covid* or coronavirus* or corona* virus* or Cov or "SARS-CoV-2*" or "SARSCoV-2*" or "SARSCoV2*" or "SARS-CoV2*" or "severe acute respiratory syndrome*" or Ncov* or "n-cov")).ti,ab.	289
**27**	((ongoing* or endur* or long* or legacy* or slow* or gradual* or protract* or lengthy* or chronic* or persist* or relaps* or remit* or remission* or residual* or delay* or prolong* or extend* or linger* or permanent* or fluctuat* or multisystem* or "multi system*" or nonrecover* or "non recover*" or subacute* or "sub acute*" or lasting* or continuous* or continual* or continuing* or postacute* or "post acute*" or postdischarg* or "post discharg*" or postinfect* or "post infect*" or postviral* or "post viral*" or postvirus* or "post virus*" or "medium* term*" or mediumterm*) adj3 ((physiolog* or neuro* or cardio* or gastro* or musculo* or renal* or kidney* or cognitive* or cognition* or rheumato* or dermatol* or skin* or haematol* or blood* or autonomic* or nervous* or nervous system* or otolaryngol* or laryngol* or otolog* or cerebro* or brain* or vascular* or respirator* or lung* or pulmonary* or psycholog* or mental health* or mental* or psychiatr* or exertion* or debilit* or devitali* or enervat* or drain* or sleep* or weak* or tired* or frail* or sapp* or strength* or confusion* or letharg* or fatigue* or tired* or weariness* or exhaust* or malaise* or pain* or headache* or breathless* or breathing* or myalgia* or delirious* or delirium* or appetite* or muscle* or muscular* or fitness* or memory* or stress* or depress* or anxiety* or emotion* or cough* or fever* or temperatur* or pneumon* or conjunctivit* or throat* or pharyngit* or dyspnea* or dyspnoea* or sick* or nausea* or nauseous* or vomit* or diarrhoea* or diarrhea* or taste* or anosmia* or smell* or olfact* or sweat* or dehydrat* or pyrexi* or nasal* or nose* or mucus* or ear* or hearing* or deaf* or "brain fog*" or cardiac* or thoracic* or chest* or ischemic* or ischaemic* or heart* or liver* or hepatic* or immuno* or palpitation* or vertigo* or metabol* or vestibular* or endocrine* or encephalit* or physical* or cough* or fibrosis* or myocarditis* or Guillain* or barre* or neuralgi* or amyotroph* or thrombo* or clot* or rash* or hive* or urticari* or lymph* or stroke* or TIA or toe* or foot* or feet* or finger* or chilblain* or numb* or inflammat* or	559

	inflame* or arthralgi* or eye* or organ or organs or tingl* or sting* or burn* or bladder* or urogenit* or genitourin* or genital* or reproducti* or urinary* or joint* or tachycard* or atrial* or autoimmun* or dysautonomi* or polyneuro* or mast* or mobility* or walking* or ambulation* or energy* or dizzy* or dizziness* or concentration* or abdominal* or abdomen* or anorexia* or anorexic* or sore* or tinnitus* or arrhythmia*) adj1 (sequela* or complication* or consequence* or effect* or event* or impact* or outcome* or reaction* or complexit* or aftercare* or impair* or problem* or issue* or symptom* or disorder* or abnormal*)) adj10 (covid* or coronavirus* or corona* virus* or Cov or "SARS-CoV-2*" or "SARSCoV-2*" or "SARSCoV2*" or "SARS-CoV2*" or "severe acute respiratory syndrome*" or Ncov* or "n-cov")).ti,ab.	
**28**	((physiolog* or neuro* or cardio* or gastro* or musculo* or renal* or kidney* or cognitive* or cognition* or rheumato* or dermatol* or skin* or haematol* or blood* or autonomic* or nervous* or nervous system* or otolaryngol* or laryngol* or otolog* or cerebro* or brain* or vascular* or respirator* or lung* or pulmonary* or psycholog* or mental health* or mental* or psychiatr* or exertion* or debilit* or devitali* or enervat* or drain* or sleep* or weak* or tired* or frail* or sapp* or strength* or confusion* or letharg* or fatigue* or tired* or weariness* or exhaust* or malaise* or pain* or headache* or breathless* or breathing* or myalgia* or delirious* or delirium* or appetite* or muscle* or muscular* or fitness* or memory* or stress* or depress* or anxiety* or emotion* or cough* or fever* or temperatur* or pneumon* or conjunctivit* or throat* or pharyngit* or dyspnea* or dyspnoea* or sick* or nausea* or nauseous* or vomit* or diarrhoea* or diarrhea* or taste* or anosmia* or smell* or olfact* or sweat* or dehydrat* or pyrexi* or nasal* or nose* or mucus* or ear* or hearing* or deaf* or "brain fog*" or cardiac* or thoracic* or chest* or ischemic* or ischaemic* or heart* or liver* or hepatic* or immuno* or palpitation* or vertigo* or metabol* or vestibular* or endocrine* or encephalit* or physical* or cough* or fibrosis* or myocarditis* or Guillain* or barre* or neuralgi* or amyotroph* or thrombo* or clot* or rash* or hive* or urticari* or lymph* or stroke* or TIA or toe* or foot* or feet* or finger* or chilblain* or numb* or inflammat* or inflame* or arthralgi* or eye* or organ or organs or tingl* or sting* or burn* or bladder* or urogenit* or genitourin* or genital* or reproducti* or urinary* or joint* or tachycard* or atrial* or autoimmun* or dysautonomi* or polyneuro* or mast* or mobility* or walking* or ambulation* or energy* or dizzy* or dizziness* or concentration* or abdominal* or abdomen* or anorexia* or anorexic* or sore* or tinnitus* or arrhythmia*) adj3 (postcovid* or post covid* or postcoronavirus* or postcorona* virus* or post coronavirus* or post corona* virus* or postcoronavirinae* or postcorona* virinae* or post coronavirinae* or post corona* virinae* or postCov or post Cov or postsars* or post sars* or "post severe acute respiratory syndrome*" or postncov* or post ncov*)).ti,ab.	599
**29**	((physiolog* or neuro* or cardio* or gastro* or musculo* or renal* or kidney* or cognitive* or cognition* or rheumato* or dermatol* or skin* or haematol* or blood* or autonomic* or nervous* or nervous system* or otolaryngol* or laryngol* or otolog* or cerebro* or brain* or vascular* or respirator* or lung* or pulmonary* or psycholog* or mental health* or mental* or psychiatr* or exertion* or debilit* or devitali* or enervat* or drain* or sleep* or weak* or tired* or frail* or sapp* or strength* or confusion* or letharg* or fatigue*	2,453

	or tired* or weariness* or exhaust* or malaise* or pain* or headache* or breathless* or breathing* or myalgia* or delirious* or delirium* or appetite* or muscle* or muscular* or fitness* or memory* or stress* or depress* or anxiety* or emotion* or cough* or fever* or temperatur* or pneumon* or conjunctivit* or throat* or pharyngit* or dyspnea* or dyspnoea* or sick* or nausea* or nauseous* or vomit* or diarrhoea* or diarrhea* or taste* or anosmia* or smell* or olfact* or sweat* or dehydrat* or pyrexi* or nasal* or nose* or mucus* or ear* or hearing* or deaf* or "brain fog*" or cardiac* or thoracic* or chest* or ischemic* or ischaemic* or heart* or liver* or hepatic* or immuno* or palpitation* or vertigo* or metabol* or vestibular* or endocrine* or encephalit* or physical* or cough* or fibrosis* or myocarditis* or Guillain* or barre* or neuralgi* or amyotroph* or thrombo* or clot* or rash* or hive* or urticari* or lymph* or stroke* or TIA or toe* or foot* or feet* or finger* or chilblain* or numb* or inflammat* or inflame* or arthralgi* or eye* or organ or organs or tingl* or sting* or burn* or bladder* or urogenit* or genitourin* or genital* or reproducti* or urinary* or joint* or tachycard* or atrial* or autoimmun* or dysautonomi* or polyneuro* or mast* or mobility* or walking* or ambulation* or energy* or dizzy* or dizziness* or concentration* or abdominal* or abdomen* or anorexia* or anorexic* or sore* or tinnitus* or arrhythmia*) adj1 (sequela* or complication* or consequence* or complexit*) adj10 (covid* or coronavirus* or corona* virus* or Cov or "SARS-CoV-2*" or "SARSCoV-2*" or "SARSCoV2*" or "SARS-CoV2*" or "severe acute respiratory syndrome*" or Ncov* or "n-cov")).ti,ab.	
**30**	(((((multi or multiple* or overlap* or cluster* or numerous* or varied* or variety*) adj1 (symptom* or system* or disease* or disorder* or illness* or condition* or syndrome*)) or (multisymptom* or multisystem* or multidisease* or multidisorder* or multiillness* or multicondition* or multisyndrom*)) adj10 (covid* or coronavirus* or corona* virus* or Cov or "SARS-CoV-2*" or "SARSCoV-2*" or "SARSCoV2*" or "SARS-CoV2*" or "severe acute respiratory syndrome*" or Ncov* or "n-cov")) not ("Multisystem* inflammatory* syndrome*" or "inflammatory* multisystem* syndrome*")).ti,ab.	526
**31**	or/20-30	6,398
**32**	7 or 31	10,175
**33**	(physiotherapist* or (occupational adj therapist*) or (physical adj therapist*) or "medical professional*" or doctor* or physician* or clinician* or nurs* or midwif* or midwives or (health adj (professional* or worker* or staff* or practitioner*)) or (healthcare adj (professional* or worker* or staff* or practitioner*)) or paramedic* or "care professional*" or "front line").mp.	1,792,932
**34**	exp Health Personnel/	573,291
**35**	(radiographer* or "allied health professional*" or AHP or AHPs).mp.	7,273
**36**	((nhs or hospital) adj (staff or worker* or professional* or practitioner*)).mp.	8,189
**37**	exp medical staff/ or exp nursing staff/	93,971

**38**	((clean* or porter* or domestic) adj (staff or worker*)).mp.	707
**39**	(non-clinical adj (staff or worker* or workforce)).mp.	237
**40**	exp hospital personnel/	94,019
**41**	"healthcare assistant*".mp.	573
**42**	(cleaner* or porter*).mp.	7,817
**43**	Food Service, Hospital/	4,833
**44**	((hospital or healthcare or health) adj5 (porter* or clean* or domestic* or food* or catering* or cafeteria or canteen or restaurant) adj3 (staff or worker* or workforce)).mp.	518
**45**	or/33-44	1,995,021
**46**	32 and 45	1,366
**47**	limit 46 to yr=”2019 – 2022”	1,354

Supplemental Material 3. Characteristics of include studies

Author and year of publication	Study design	Country	Setting/context	Time period	sample size (N)	Female n (%)	Mean age (SD)	Population	Main outcomes/measures	Follow-up period	COVID-19 infection confirmed by	Long COVID criteria	Mention of ethnicity	Critical Appraisal Score (%)

**Fijałkowska, Jadwiga, et al. (2021)**	**Short communication**	**Poland**	**Hospital**	**Dec 2020 - Apr 2021**	**89**	**69 (77)**	**42 (10)**	**HCWs who recovered from non-severe COVID-19**	**Myocardial injury**	**4 months**	**PCR**	**NA**		**56**
**Navarro-Font, Xavier, et al. (2021)**	**Retrospective cohort**	**Spain**	**Hospital**	**Sep 2020 - Oct 2020**	**119**	**(78.3)**	**40.6 (11.6)**	**HCWs from CUN**	**Severity of COVID-19 and chronic disease sequelae**	**4-6 months**	**PCR**	**NA**		**89**
**Glück, Vivian, et al. (2021)**	**Prospective cohort**	**Germany**	**Hospital**	**Apr 2020 - Dec 2020**	**123**	**75 (62)**	**Median (Females=37, Males=40)**	**Infected HCWs from a network of 6 hospitals in the southeast region of Germany**	**Antibody status**	**30 week (7.5 months)**	**PCR**	**NA**		**78**

**Tawfik, Heba Mohamed, et al. (2021)**	**Retrospective cohort**	**Egypt**	**Ain-Shams University, the MOH and population hospitals**	**NA**	**120**	**70 (58 %)**	**33.7 (7.3)**	**HCWs (i.e., doctors, nurses, dentists, pharmacists)**	**Symptoms of long COVID**	**1-3 months**	**PCR**	**Persistence symptoms at 3 months**		**44**
**Havervall, Sebastian, et al. (2021)**	**Prospective cohort**	**Sweden**	**Danderyd Hospital**	**Apr 2020 - May 2020**	**323**	**268 (83 %)**	**Median (IQR) 43 (33-52)**	**HCWs who consented to fill out the questionnaires and do blood sampling every 4 months**	**Sheehan Disability Scale and symptoms**	**8 months**	**NA**	**Persistent symptoms at 4 months**		**78**

**Rao, Shwethapriya, et al. (2021)**	**Cross-sectional**	**India**	**Web-based survey**	**Jan 2021 - Mar 2021**	**163**	**(59)**	**NA**	**HCWs (i.e., doctors, nurses, allied health professionals)**	**Post-COVID-19 syndromes**	**3 months**	**NA**	**Persistent symptoms following recovery from COVID-19 (duration not mentioned)**		**44**
**Strahm, Carol, et al. (2022)**	**Prospective cohort**	**Switzerland**	**Hospital**	**Jul 2020 - Jan 2021**	**556**	**453 (81)**	**Median (IQR) 38.9 (16.8-63.5)**	**HCWs (i.e., nurses, physicians, others) from 23 healthcare institutions in northern and eastern Switzerland**	**Symptom questionnaire associated with long COVID and 4 psychometric scores**	**Different ranges up to 24 weeks**	**PCR & antibody**	**Persistence of symptoms 4 weeks after acute infection**	**Caucasian-775/784 (99%), Other-9/784 (1.1%)**	**78**

**Eiros, Rocío, et al. (2021)**	**Cross-sectional**	**Spain**	**Hospital**	**May 2020 - Jun 2020**	**139**	**100 (72)**	**Median (IQR) 52 (41-57)**	**HCWs from University Hospital of Salamanca**	**CMR images and immunophenotypic analysis of peripheral blood sample**	**10.4 weeks**	**PCR & antibody**	**NA**		**78**
**Liao, Tingting, et al. (2021)**	**Prospective cohort**	**China**	**Hospital**	**Mar 2021 - Apr 2021**	**210**	**171 (81)**	**Median (IQR) 38 (33-48)**	**HCWs working in an outpatient clinic of the Wuhan Union Hospital.**	**Six min walk test, physical and psychiatric measures for PTSD**	**1 year**	**NA**	**NA**		**89**
**Vimercati, Luigi, et al. (2021)**	**Retrospective cohort**	**Italy**	**Hospital**	**Mar 2020 - Mar 2021**	**352**	**NA**	**45.4 (13.2)**	**HCWs (i.e., nusres, doctors, HCA) working in University Hospital of Bari**	**Long COVID 34 days off work or more**	**1 year**	**PCR**	**Being off work for >34 days**		**33**

**Sultana, Sarmin, et al. (2021)**	**Cross-sectional**	**Bangladesh**	**Foundation**	**Apr 2020 - Jul 2020**	**186**	**63 (33.9)**	**34.8 (9.9)**	**Bangladesh medical doctors in Bangladesh Doctors Foundation**	**Long COVID symptoms**	**2 months**	**PCR**	**At least one of long COVID symptoms persisting > 60 days**		**22**
**Kisiel, Marta, et al. (2021)**	**Prospective cohort**	**Sweden**	**Hospital**	**Mar 2020 - Aug 2020**	**433**	**335 (77)**	**42 (12)**	**Symptomatic employees of The Department of Infectious Disease at the Uppsala University who did not receive inpatient care**	**Days of sick leave**	**8-12 months**	**PCR**	**Sick leave of >3 weeks**		**78**

Martinez , Aurelien Emmanuel , et al. (2021)	Retrospective cohort	Switzerland	Hospital	Apr 2021- Jun 2021	260	196 (75.4)	Median (IQR) 37 (28-49)	HCWs at University Hospital Basel who replied to the online questionnaire following their COVID-19 infection	Persistent COVID-19 symptoms	3-12 months	Self-reported	Persistent symptoms for ≥ 3 months		78
Abu-Hammad , Osama, et al. (2021)	Cross-sectional	Saudi Arabia	Dental hospital	Mar 2021 - Aug 2021	62	30 (17.3)	NA	Clinical dental students (4th, 5th, 6th years, and interns) and faculty currently practicing at the hospital	Types of long-term health and socioeconomic complications among infected personnel	NA	NA	NA		56

Gaber, T A-Z K, et al. (2021)	Cross-sectional	UK	Web-based survey	Jul 2020 - Aug 2020	138	127 (92)	NA	HCWs who work in Wrightington, Wigan and Leigh NHS Teaching Trust	Post-COVID-19 symptoms with post-viral fatigue	4 months	Antibody	NA		-
Xiong, Li-Juan, et al. (2021)	Case-control	China	Web-based survey	Jul 2020 - NA	291	236 (81.1)	37.3 (8.9)	HCWs who were recovered at the time of the study.	Risk of PTSD	6 months	PCR	NA		67
Sawant, N, et al. (2021)	Cross-sectional	India	Web-based survey	Oct 2020 - end of Oct 2020	79	33 (41.8)	26.4 (3.3)	Resident doctors and interns from the hospital database of COVID affected healthcare personnel who were completely recovered.	Presence of anxiety and depressive symptoms	4 months		NA		44

Dudine, L, et al. (2021)	Cross-sectional	Italy	Community	Jun 2020 - Sep 2020	104	71 (68.3)	42 (11)	HCW of the Trieste area in Northern Italy	The association between olfactory and gustatory dysfunctions and a state of distress	NA	NA	NA		67
Nielsen, KJ, et al. (2021)	Prospective cohort	Denmark	All hospitals in the Central Denmark region	Apr 2020 - Jun 2020	210	177 (84.3)	IQR=(40-49)	HCWs (i.e., nursing staff, medical doctors, biomedical laboratory scientists & medical secretaries)with COVID-19 during data collection period	Symptom profile	90 days (three time periods (0–30, 31–60, and 61–90 days))	PCR	60-90 days post positive SARS-CoV-2 test used		89

**Kinge, Constance Wose, et al. (2022)**	**Cross-sectional**	**South Africa**	**Web-based survey**	**Feb 2021 - Apr 2021**	**62**	**47 (76)**	**Median IQR 33.5 (30–44)**	**NGO workers in one of seven provinces who had COVID-19 and are fighting HIV & AIDS**	**Long COVID prevalence among HIV & AIDS NGO workers**	**3 months**	**PCR**	**Post-acute COVID defined as per protocol**	**African, Caucasian & coloured**	**89**
**Bozan, Öner, et al. (2021)**	**Cross-sectional**	**Turkey**	**Web-based survey**	**1 Mar 2021 - 15 Mar 2021**	**248**	**130 (52.4)**	**32.8 (8.0)**	**HCWs who were infected with COVID-19 and recovered at least 30 days before data collection.**	**differences or changes in the quality of sleep of patients recovering from COVID-19 infection**	**NA**	**PCR**	**NA**		**44**

Bussière, Nicholas, et al. (2021)	Cross-sectional	Canada	Web-based survey	Aug 2020 - Oct 2020	704	111 (15.8)	42 (11.7)	HCWs from Quebec who took part in a study from the Institut National de Santé Publique du Québec and agreed to be re-contacted for further research.	Differences in reported chemosensory self-assessment of olfactory, gustatory, and trigeminal functions across time points and Chemosensory Perception Test scores	3-7 months	PCR	This study reports chemosensory dysfunction 3-7 months following SARS-CoV-2 infection in RT-PCR confirmed healthcare workers. The questionnaire was completed on average 4.8 (SD: 0.8, range 3-7) months after symptom onset.		56

**Uvais, Nalakath A., et al. (2021)**	**Cross-sectional**	**India**	**Hopsital**	**NA**	**107**	**(63. 6)**	**27.5**	**HCWs from a tertiary care hospital in South India**	**Insomnia and its severity**	**NA**	**Self-report ed**	**NA**		**56**

**Mattioli, Flavia, et al. (2021)**	**Prospective cohort**	**Italy**	**Hospital**	**NA**	**120**	**(70)**	**47.86**	**HCWs who worked at The Unit of Occupational Health of the general University Hospital of Brescia who underwent a targeted clinical diagnostic assessment including, a neurological exam and a detailed neuropsychological evaluation**	**Health status after COVID-19**	**4 months**	**PCR**	**4 month following positive RT-PCR**		**78**

**Tempany, M, et al. (2021)**	**Cross-sectional**	**Ireland**	**Hospital**	**Jun 2020 - Nov 2020**	**217**	**174 (80.2)**	**NA**	**HCWs who presented for SARS-CoV-2 antibody testing at least 12 weeks since confirmed infection or onset of symptoms**	**Degree of recovery & nature of persistent symptoms**	**At least 12 weeks**	**PCR & antibody**	**Persistent symptoms for 12 weeks**		**78**
	**Gray Literature (pre-prints)**													
**Dennis, A, et al. (Pre-print)**	**Prospective cohort**	**UK**	**Two non-acute settings**	**Apr 2020 - Aug 2021**	**172**	**142 (82.5)**	**43.96 (10.9)**	**HCWs in Oxford and London with evidence of organ impairment following recovery from acute COVID-19**	**Prevalence of single and multi-organ impairment**	**At 6 and 12 months**	**PCR & antibody**		**White 145, mixed 6, south Asian 15 , Black 6**	**78**

**Gruber, R, et al.(Pre-print)**	**Retrospective cohort**	**Germany**	**Medical centers**	**Jun 2021 - Oct 2021**	**221**	**158 (71.5)**	**39.8 (12.5)**	**HCWs (nursing staff, medicals taff, administration, cleaning staff and others) with severe acute infection from three medical centers in Cologne, Germany.**	**Frequency and duration of long-term COVID-19 symptoms**	**491 days with six-weeks intervals**	**PCR & antibody**		**NA**	**78**

**Mohr, N M,et al (Pre-print)**	**Nested cohort**	**USA**	**Academic medical centers**	**Dec 2020 - Aug 2021**	**419**	**352 (84)**	**38.4 (10.6)**	**HCWs (non-clinical, physicians, nursing professionals, housekeeping) who were working on-site in 12 U.S states**	**Presence of symptoms 6 weeks after onset of COVID-19 illness and days to return to work after COVID-19 illness**	**six weeks**	**PCR & antibody**		**White non-hispanic 303(72.3), Black non-hispanic 47 (11.2), Hispanic or Latino 41 (9.8), Other non-hispanic 28 (6.7)**	**89**

Supplemental Material 4. Critical appraisal of included studies

Author and year of publication	Q1	Q2	Q3	Q4	Q5	Q6	Q7	Q8	Q9	(%) Yes
Fijałkowska, Jadwiga, et al. (2021)	N	U	N	Y	Y	Y	Y	Y	U	56
Navarro-Font, Xavier, et al. (2021)	Y	Y	N	Y	Y	Y	Y	Y	Y	89
Glück, Vivian, et al. (2021)	Y	Y	Y	Y	N	Y	Y	Y	U	78
Tawfik, Heba Mohamed, et al. (2021)	Y	Y	Y	N	N	Y	N	N	N	44
Havervall, Sebastian, et al. (2021)	Y	Y	N	Y	N	Y	Y	Y	Y	78
Rao, Shwethapriya, et al. (2021)	Y	Y	N	Y	N	N	N	N	Y	44
Strahm, Carol, et al. (2022)	Y	Y	Y	Y	N	Y	Y	Y	N	78
Eiros, Rocío, et al. (2021)	Y	Y	N	Y	Y	Y	Y	Y	N	78
Liao, Tingting, et al. (2021)	Y	Y	N	Y	Y	Y	Y	Y	Y	89
Vimercati, Luigi, et al. (2021)	Y	N	Y	Y	N	N	N	N	N	33
Sultana, Sarmin, et al. (2021)	N	N	Y	Y	N	N	N	N	N	22
Kisiel, Marta, et al. (2021)	Y	Y	Y	Y	Y	N	N	Y	Y	78
Martinez, Aurelien Emmanuel , et al. (2021)	Y	Y	N	Y	Y	Y	Y	N	Y	78
Abu-Hammad, Osama, et al. (2021)	Y	Y	Y	N	U	N	Y	U	Y	56

**Gaber, T A-Z K, et al. (2021)**	**Correspondence letter**									
**Xiong, Li-Juan, et al. (2021)**	**Y**	**Y**	**N**	**Y**	**Y**	**Y**	**NA**	**Y**	**N**	**67**
**Sawant, N, et al. (2021)**	**Y**	**Y**	**N**	**Y**	**N**	**Y**	**N**	**N**	**N**	**44**
**Dudine, L, et al. (2021)**	**Y**	**U**	**Y**	**Y**	**Y**	**Y**	**Y**	**N**	**U**	**67**
**Nielsen, KJ, et al. (2021)**	**Y**	**Y**	**Y**	**Y**	**Y**	**Y**	**Y**	**Y**	**U**	**89**
**Kinge, Constance Wose, et al. (2022)**	**Y**	**Y**	**Y**	**Y**	**Y**	**Y**	**Y**	**Y**	**U**	**89**
**Bozan, Öner, et al. (2021)**	**Y**	**Y**	**U**	**N**	**Y**	**U**	**U**	**Y**	**U**	**44**
**Bussière, Nicholas, et al. (2021)**	**Y**	**Y**	**Y**	**N**	**Y**	**N**	**U**	**N**	**Y**	**56**
**Uvais, Nalakath A., et al. (2021)**	**Y**	**U**	**Y**	**Y**	**N**	**Y**	**Y**	**N**	**N**	**56**
**Mattioli, Flavia, et al. (2021)**	**Y**	**Y**	**Y**	**N**	**Y**	**Y**	**U**	**Y**	**Y**	**78**
**Tempany, M, et al. (2021)**	**N**	**Y**	**Y**	**N**	**Y**	**Y**	**Y**	**Y**	**Y**	**78**
**Grey Literature (Pre-prints)**										
**Dennis, A, et al. (Pre-print)**	**Y**	**Y**	**Y**	**Y**	**Y**	**Y**	**U**	**N**	**Y**	**78**
**Gruber, R, et al.(Pre-print)**	**Y**	**Y**	**Y**	**Y**	**Y**	**N**	**Y**	**N**	**Y**	**78**
**Mohr, N M,et al (Pre-print)**	**Y**	**Y**	**Y**	**Y**	**Y**	**Y**	**U**	**Y**	**Y**	**89**
**Total (%) Yes**	**86**	**79**	**61**	**75**	**61**	**68**	**54**	**54**	**46**	

Y*: Yes;* **U***: Unclear;* **N***: No;* **NA***: Not Available*

JBI critical appraisal questions:
Q1. *Was the sample frame appropriate to address the target population?*
Q2*. Were study participants sampled in an appropriate way*
Q3*. Was the sample size adequate?*
Q4. *Were the study subjects and the setting described in detail?*
Q5*. Was the data analysis conducted with sufficient coverage of the identified sample?*
Q6*. Were valid methods used for the identification of the condition?*

Q7. *Was the condition measured in a standard, reliable way for all participants?*
Q8. *Was there appropriate statistical analysis?*
Q9. *Was the response rate adequate, and if not, was the low response rate managed appropriately?*

Supplemental Material 5. DOI plot for Publication bias

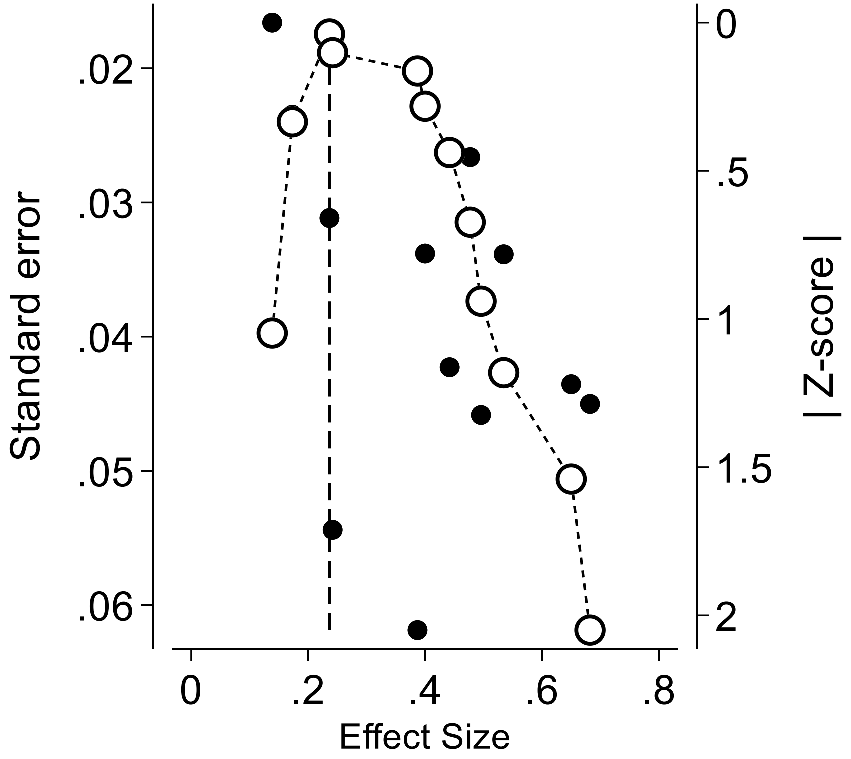

LFK index = 3.63; Egger's test (p_value) = .011

***Number of included studies = 12**

Supplemental Material 6. COVID-19 HCWs survivors – Sub-group analysis by region-

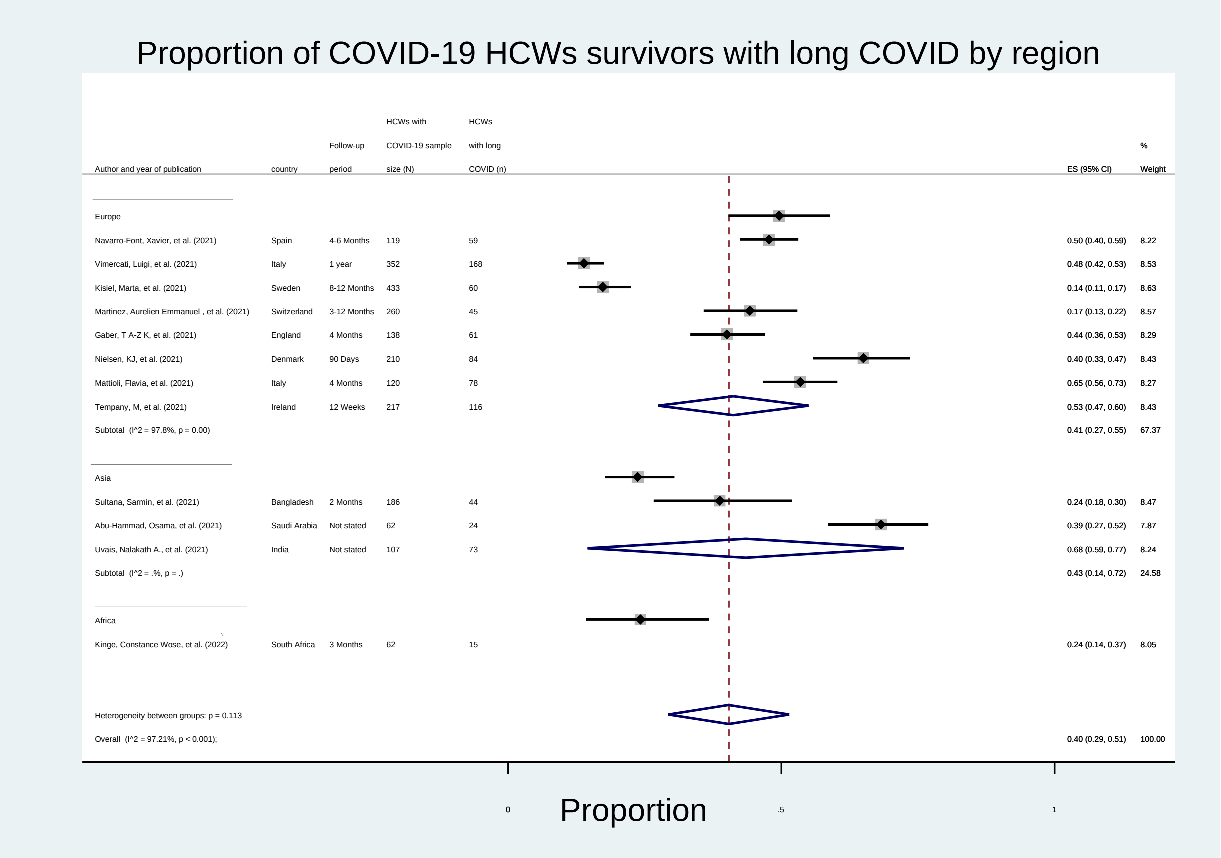

Supplemental Material 7. Forest plot showing point estimates with 95% confidence intervals for prevalence of the remaining six most common long COVID symptoms among COVID-19 HCWs survivors

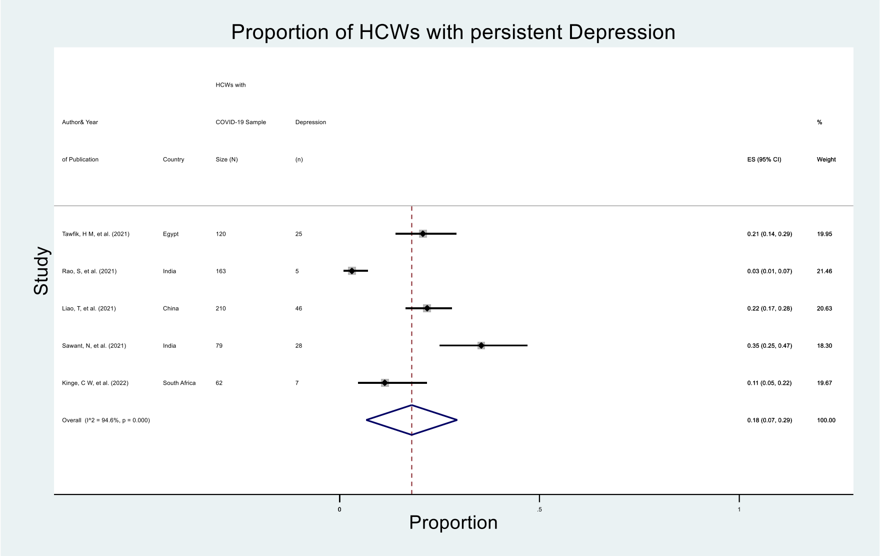
Proportion of HCWs with persistent Depression
Study
HCWs with
Author& Year
COVID-19 Sample
Depression
%
of Publication
Country
Size (N)
(n)
ES (95% CI)
Weight
Tawfik, H M, et al. (2021) Egypt 120 25 0.21 (0.14, 0.29) 19.95
Rao, S, et al. (2021) India 163 5 0.03 (0.01, 0.07) 21.46
Liao, T, et al. (2021) China 210 46 0.22 (0.17, 0.28) 20.63
Sawant, N, et al. (2021) India 79 28 0.35 (0.25, 0.47) 18.30
Kinge, C W, et al. (2022) South Africa 62 7 0.11 (0.05, 0.22) 19.67
Overall (I^2 = 94.6%, p = 0.000) 0.18 (0.07, 0.29) 100.00
0
.5
1
Proportion

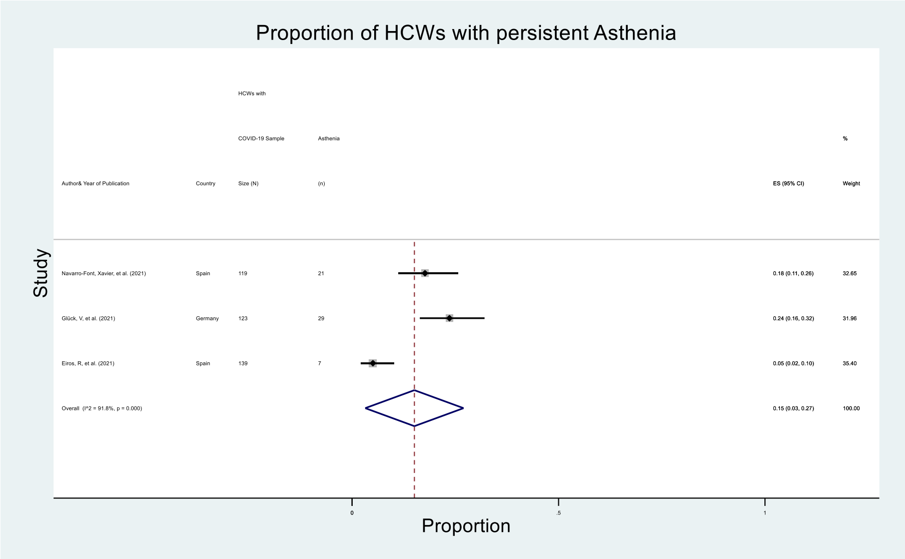
Proportion of HCWs with persistent Asthenia
Study
HCWs with
COVID-19 Sample
Asthenia
%
Author& Year of Publication
Country
Size (N)
(n)
ES (95% CI)
Weight
Navarro-Font, Xavier, et al. (2021) Spain 119 21 0.18 (0.11, 0.26) 32.65
Glück, V, et al. (2021) Germany 123 29 0.24 (0.16, 0.32) 31.96
Eiros, R, et al. (2021) Spain 139 7 0.05 (0.02, 0.10) 35.40
Overall (I^2 = 91.8%, p = 0.000) 0.15 (0.03, 0.27) 100.00
0
.5
1
Proportion

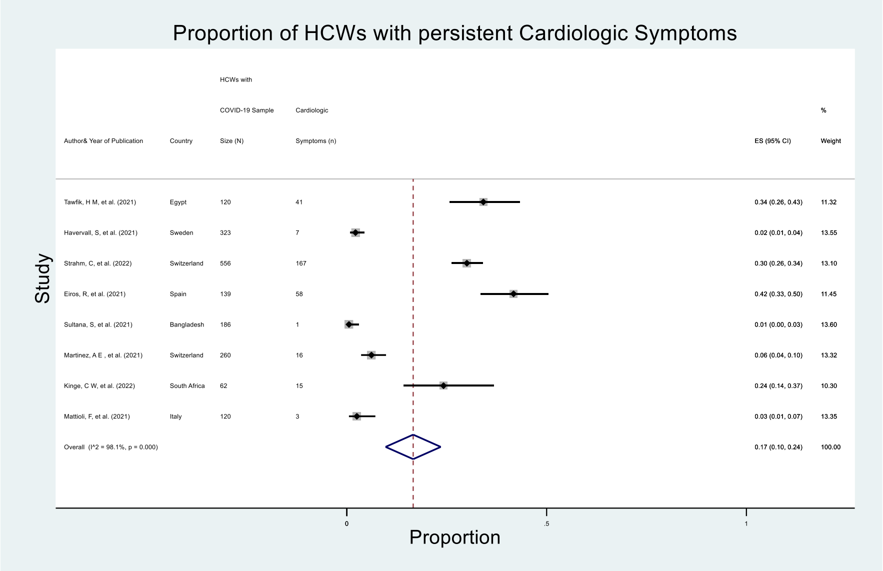
Proportion of HCWs with persistent Cardiologic Symptoms
Study
HCWs with
COVID-19 Sample
Cardiologic
%
Author& Year of Publication
Country
Size (N)
Symptoms (n)
ES (95% CI)
Weight
Tawfik, H M, et al. (2021) Egypt 120 41 0.34 (0.26, 0.43) 11.32
Havervall, S, et al. (2021) Sweden 323 7 0.02 (0.01, 0.04) 13.55
Strahm, C, et al. (2022) Switzerland 556 167 0.30 (0.26, 0.34) 13.10
Eiros, R, et al. (2021) Spain 139 58 0.42 (0.33, 0.50) 11.45
Sultana, S, et al. (2021) Bangladesh 186 1 0.01 (0.00, 0.03) 13.60
Martinez, A E , et al. (2021) Switzerland 260 16 0.06 (0.04, 0.10) 13.32
Kinge, C W, et al. (2022) South Africa 62 15 0.24 (0.14, 0.37) 10.30
Mattioli, F, et al. (2021) Italy 120 3 0.03 (0.01, 0.07) 13.35
Overall (I^2 = 98.1%, p = 0.000) 0.17 (0.10, 0.24) 100.00
0
.5
1
Proportion

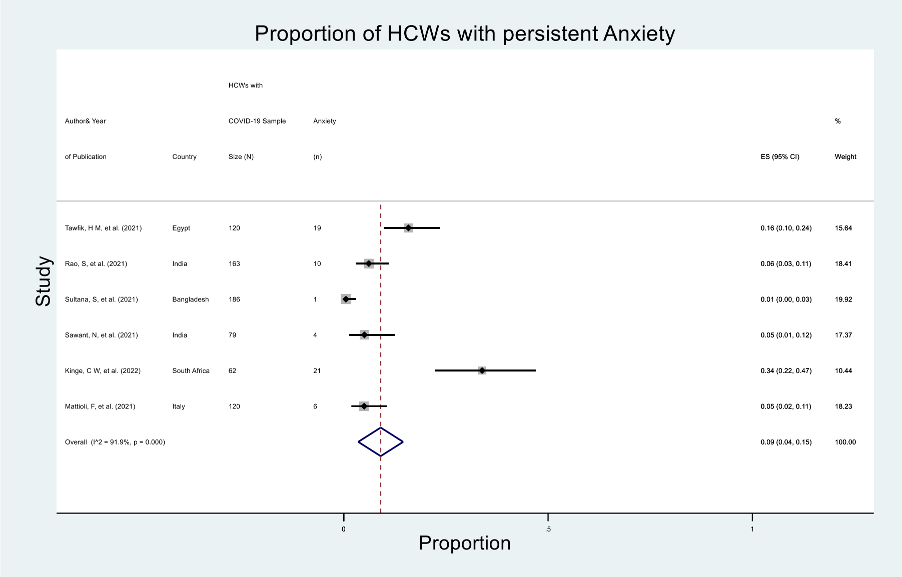
Proportion of HCWs with persistent Anxiety
Study
HCWs with
Author& Year
COVID-19 Sample
Anxiety
%
of Publication
Country
Size (N)
(n)
ES (95% CI)
Weight
Tawfik, H M, et al. (2021) Egypt 120 19 0.16 (0.10, 0.24) 15.64
Rao, S, et al. (2021) India 163 10 0.06 (0.03, 0.11) 18.41
Sultana, S, et al. (2021) Bangladesh 186 1 0.01 (0.00, 0.03) 19.92
Sawant, N, et al. (2021) India 79 4 0.05 (0.01, 0.12) 17.37
Kinge, C W, et al. (2022) South Africa 62 21 0.34 (0.22, 0.47) 10.44
Mattioli, F, et al. (2021) Italy 120 6 0.05 (0.02, 0.11) 18.23
Overall (I^2 = 91.9%, p = 0.000) 0.09 (0.04, 0.15) 100.00
0
.5
1
Proportion

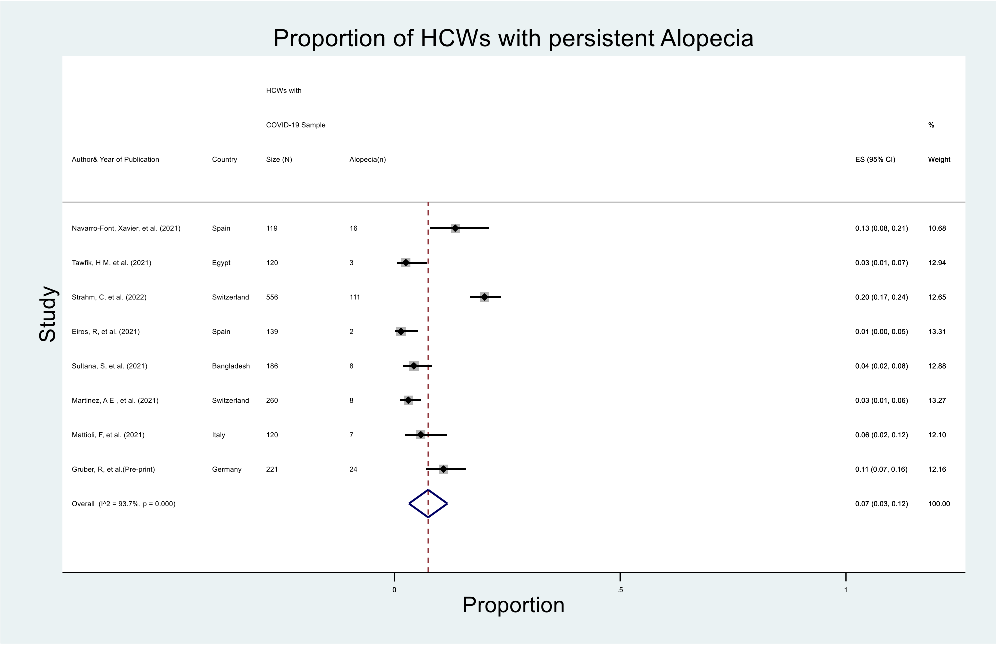
Proportion of HCWs with persistent Alopecia
Author& Year of Publication
Country
HCWs with COVID-19 Sample Size (N)
Alopecia(n)
ES (95% CI)
% Weight
Navarro-Font, Xavier, et al. (2021) Spain 119 16 0.13 (0.08, 0.21) 10.68
Tawfik, H M, et al. (2021) Egypt 120 3 0.03 (0.01, 0.07) 12.94
Strahm, C, et al. (2022) Switzerland 556 111 0.20 (0.17, 0.24) 12.65
Eiros, R, et al. (2021) Spain 139 2 0.01 (0.00, 0.05) 13.31
Sultana, S, et al. (2021) Bangladesh 186 8 0.04 (0.02, 0.08) 12.88
Martinez, A E , et al. (2021) Switzerland 260 8 0.03 (0.01, 0.06) 13.27
Mattioli, F, et al. (2021) Italy 120 7 0.06 (0.02, 0.12) 12.10
Gruber, R, et al.(Pre-print) Germany 221 24 0.11 (0.07, 0.16) 12.16
Overall (I^2 = 93.7%, p = 0.000) 0.07 (0.03, 0.12) 100.00
Study
0
.5
1
Proportion

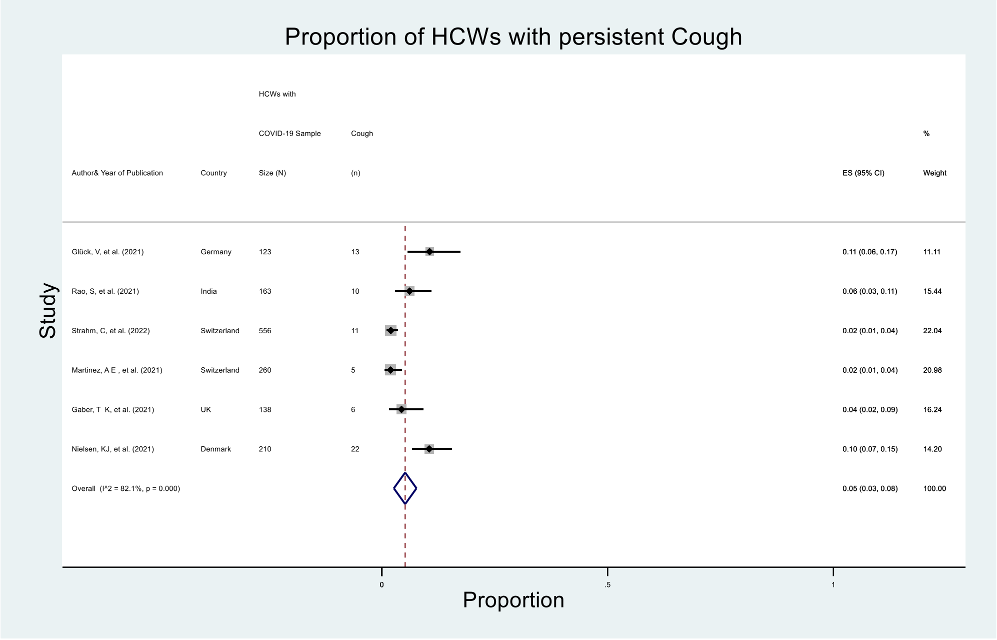
Proportion of HCWs with persistent Cough
Author& Year of Publication
Country
HCWs with COVID-19 Sample Size (N)
Cough (n)
ES (95% CI)
% Weight
Glück, V, et al. (2021) Germany 123 13 0.11 (0.06, 0.17) 11.11
Rao, S, et al. (2021) India 163 10 0.06 (0.03, 0.11) 15.44
Strahm, C, et al. (2022) Switzerland 556 11 0.02 (0.01, 0.04) 22.04
Martinez, A E , et al. (2021) Switzerland 260 5 0.02 (0.01, 0.04) 20.98
Gaber, T K, et al. (2021) UK 138 6 0.04 (0.02, 0.09) 16.24
Nielsen, KJ, et al. (2021) Denmark 210 22 0.10 (0.07, 0.15) 14.20
Overall (I^2 = 82.1%, p = 0.000) 0.05 (0.03, 0.08) 100.00
Study
0
.5
1
Proportion

Supplemental Material 8. Forest plot showing point estimates with 95% confidence intervals for prevalence of long COVID symptoms among COVID-19 HCWs survivors – Sub-group analysis by follow-up period-

***Note: two studies were removed from this analysis as they did not state the follow-up period.**

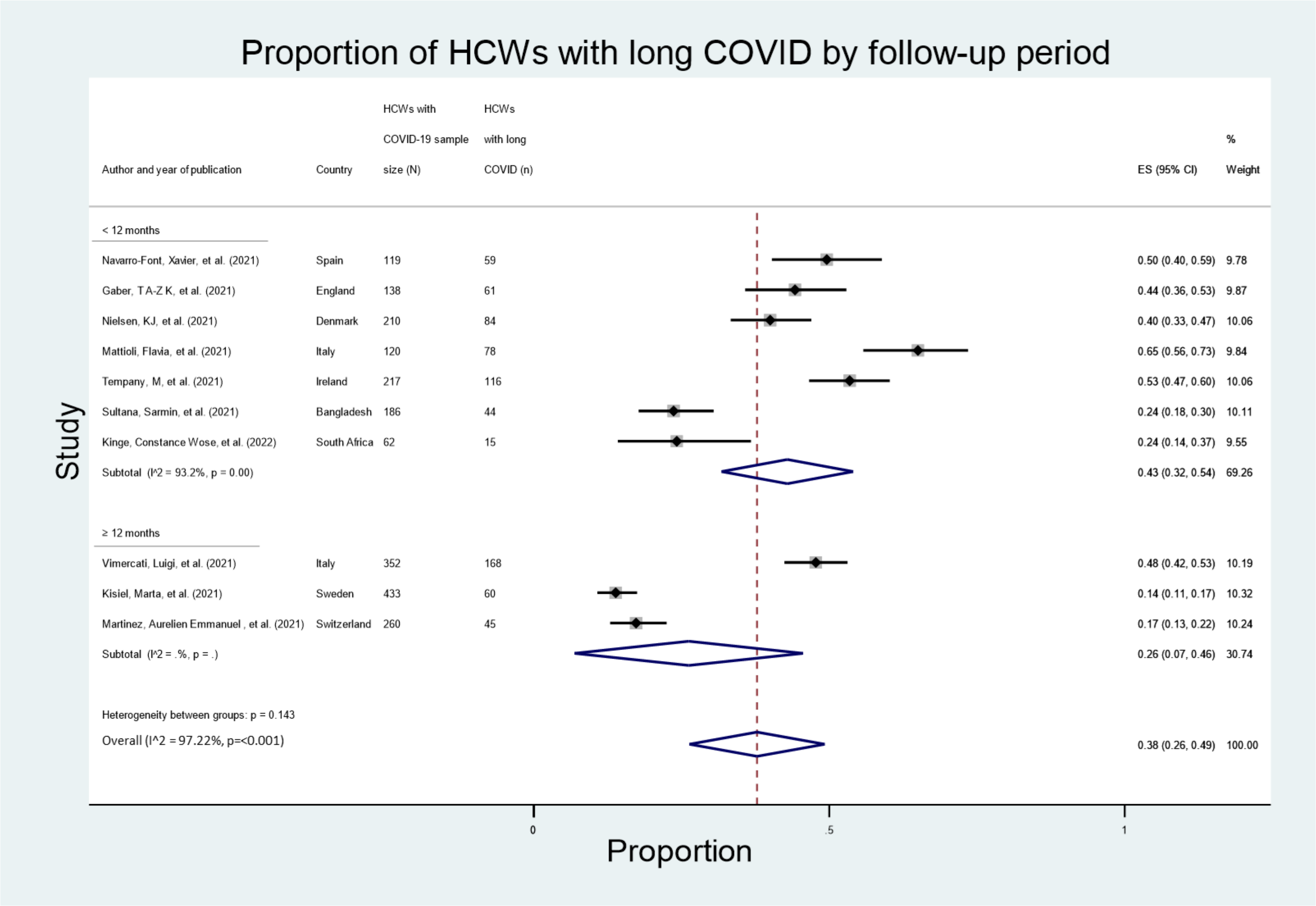
Proportion of HCWs with long COVID by follow-up period
Author and year of publication
Country
HCWs with COVID-19 sample size (N)
HCWs with long COVID (n)
ES (95% CI)
% Weight
< 12 months
Navarro-Font, Xavier, et al. (2021) Spain 119 59 0.50 (0.40, 0.59) 9.78
Gaber, TA-Z K, et al. (2021) England 138 61 0.44 (0.36, 0.53) 9.87
Nielsen, KJ, et al. (2021) Denmark 210 84 0.40 (0.33, 0.47) 10.06
Mattioli, Flavia, et al. (2021) Italy 120 78 0.65 (0.56, 0.73) 9.84
Tempany, M, et al. (2021) Ireland 217 116 0.53 (0.47, 0.60) 10.06
Sultana, Sarmin, et al. (2021) Bangladesh 186 44 0.24 (0.18, 0.30) 10.11
Kinge, Constance Wose, et al. (2022) South Africa 62 15 0.24 (0.14, 0.37) 9.55
Subtotal (I^2 = 93.2%, p = 0.00) 0.43 (0.32, 0.54) 69.26
≥ 12 months
Vimercati, Luigi, et al. (2021) Italy 352 168 0.48 (0.42, 0.53) 10.19
Kisiel, Marta, et al. (2021) Sweden 433 60 0.14 (0.11, 0.17) 10.32
Martinez, Aurelien Emmanuel , et al. (2021) Switzerland 260 45 0.17 (0.13, 0.22) 10.24
Subtotal (I^2 = .%, p = .) 0.26 (0.07, 0.46) 30.74
Heterogeneity between groups: p = 0.143
Overall (I^2 =97.22%, p=<0.001) 0.38 (0.26, 0.49) 100.00
Study
0
.5
1
Proportion

Supplemental Material 9. Sensitivity analysis to assess impact of study quality on the pooled estimated prevalence of long COVID

	All studies (regardless of the study quality)	Sensitivity analysis-after removing studies poor-quality/not peer-reviewed	Sensitivity analysis-after removing studies with self-reported data

**Pooled prevalence estimates (95% CI)**	**40% (29%-51%)**	**41% (28%-54%)**	**40% (28%-52%)**
**Number of studies**	**12 studies**	**10 studies**	**10 studies**
**I2 statistic**	**97.2%**	**97.4%**	**96.6%**

Supplemental Material 10. Forest plot showing point estimates with 95% confidence intervals for prevalence of long COVID by the most common reported symptom – Sensitivity analysis-

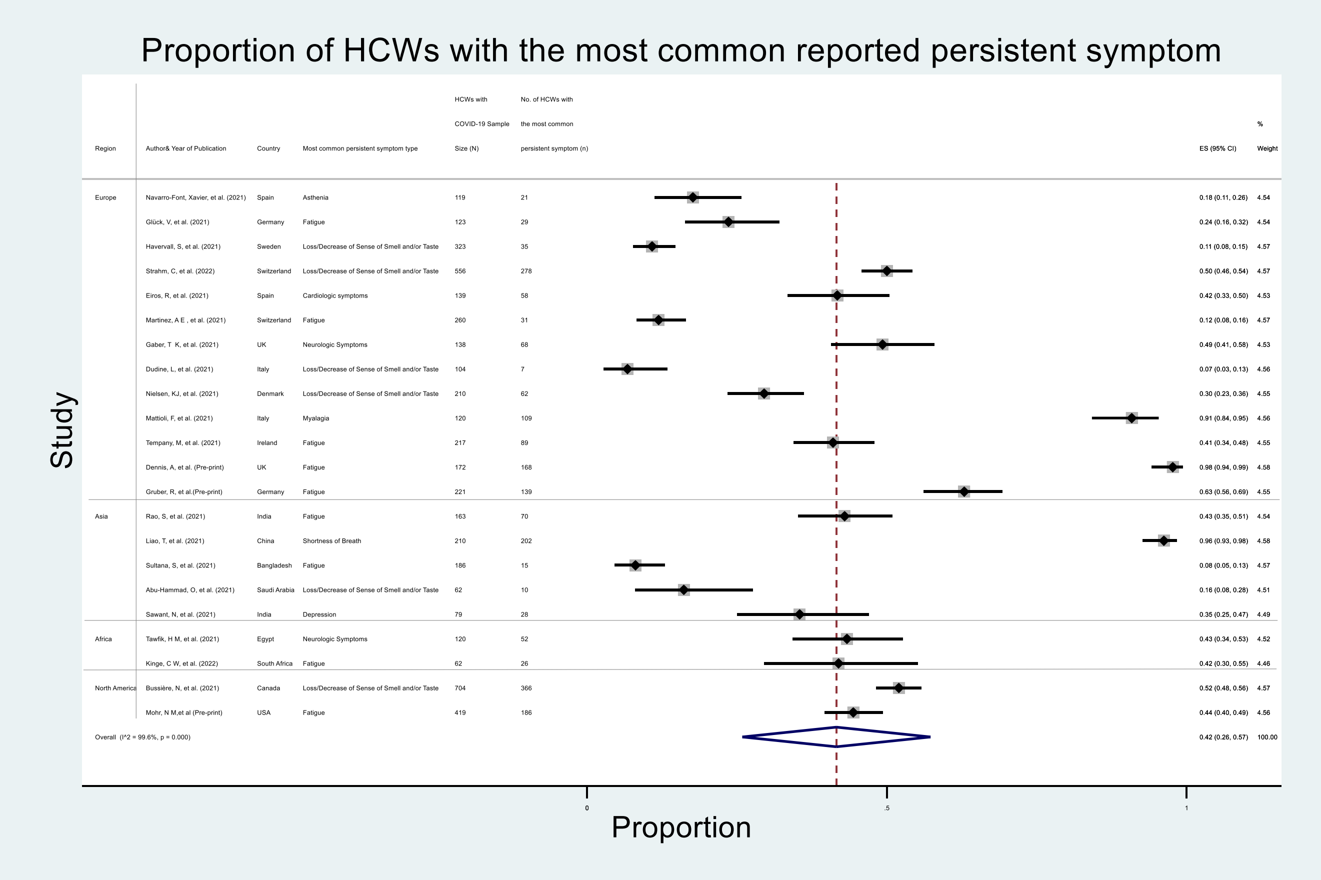

Supplement: online supplemental material 1 [file bmjph-3-1-s001.pdf]
